# Copper stress in *Staphylococcus aureus* leads to adaptive changes in central carbon metabolism†
†Electronic supplementary information (ESI) available. See DOI: 10.1039/c8mt00239h


**DOI:** 10.1039/c8mt00239h

**Published:** 2018-11-16

**Authors:** Emma Tarrant, Gustavo P. Riboldi, Matthew R. McIlvin, Jack Stevenson, Anna Barwinska-Sendra, Louisa J. Stewart, Mak A. Saito, Kevin J. Waldron

**Affiliations:** a Institute for Cell & Molecular Biosciences , Faculty of Medical Sciences , Newcastle University , Framlington Place , Newcastle upon Tyne , NE2 4HH , UK . Email: kevin.waldron@ncl.ac.uk ; Fax: +44 (0)191 208 7424 ; Tel: +44 (0)191 208 7369; b Marine Chemistry and Geochemistry Department , Woods Hole Oceanographic Institution , Woods Hole , MA 02543 , USA

## Abstract

Copper toxicity has been a long-term selection pressure on bacteria due to its presence in the environment and its use as an antimicrobial agent by grazing protozoa, by phagocytic cells of the immune system, and in man-made medical and commercial products. There is recent evidence that exposure to increased copper stress may have been a key driver in the evolution and spread of methicillin-resistant *Staphylococcus aureus*, a globally important pathogen that causes significant mortality and morbidity worldwide. Yet it is unclear how *S. aureus* physiology is affected by copper stress or how it adapts in order to be able to grow in the presence of excess copper. Here, we have determined quantitatively how *S. aureus* alters its proteome during growth under copper stress conditions, comparing this adaptive response in two different types of growth regime. We found that the adaptive response involves induction of the conserved copper detoxification system as well as induction of enzymes of central carbon metabolism, with only limited induction of proteins involved in the oxidative stress response. Further, we identified a protein that binds copper inside *S. aureus* cells when stressed by copper excess. This copper-binding enzyme, a glyceraldehyde-3-phosphate dehydrogenase essential for glycolysis, is inhibited by copper *in vitro* and inside *S. aureus* cells. Together, our data demonstrate that copper stress leads to the inhibition of glycolysis in *S. aureus*, and that the bacterium adapts to this stress by altering its central carbon utilisation pathways.

## Abstract 2

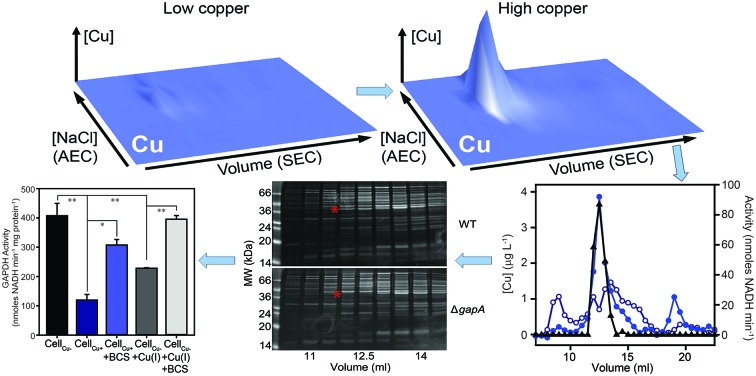
Pathogenic *Staphylococcus aureus* respond to copper stress by altering central carbon metabolism in response to a specific inhibition of the glycolytic enzyme, glyceraldehyde-3-phosphate dehydrogenase.

##

Significance to metallomics

Copper is a potent antimicrobial, and is used by the mammalian immune system to fight pathogens. Resistance to copper may have driven recent evolution of *Staphylococcus aureus*. We have used quantitative proteomics to determine the *S. aureus* adaptive response to copper, which involves induction of carbon metabolic enzymes. We then used metalloproteomic methods to identify a cytosolic protein that binds copper under stress conditions, the glycolytic enzyme glyceraldehyde-3-phosphate dehydrogenase. Together, our results demonstrate how copper stress affects central carbon metabolism in this pathogenic bacterium, and how its adaptive response to copper stress maintains this metabolism to overcome enzyme inhibition.

## Introduction

Although copper is an essential micronutrient for most organisms, required in small quantities as a cofactor in important metalloenzymes, excess copper is extremely toxic to all biological systems. The molecular mechanisms of copper toxicity are not entirely clear, but seem to involve combinations of oxidative damage caused by copper-catalysed production of reactive oxygen species (ROS), disruption of key cellular functions through strong interactions of copper ions with intracellular thiols, and its ability to bind with high affinity to metalloprotein binding sites for other essential metal ions, particularly by disrupting iron–sulphur clusters in metabolic enzymes.^1^,^2^ The relative importance of each of these mechanisms in overall copper toxicity appears to vary between organisms.^3^–^5^

Copper toxicity has likely been a constant selection pressure on organisms since the great oxidation event, when atmospheric levels of dioxygen first rose through the advent of oxygenic photosynthesis, which would have led to solubilisation of copper from previously insoluble forms through oxidative weathering of rocks.^6^ Since then, organisms have been continuously exposed to environmental copper, which has driven the evolution and selection of homeostatic systems to regulate intracellular copper, enabling its biological utilisation while simultaneously limiting its toxicity. Several components of these copper homeostasis systems are conserved between bacteria and higher eukaryotes,^7^,^8^ suggesting they are ancient in origin and that resistance to elevated copper has influenced evolution ever since.

Recent evidence has indicated that resistance to high copper may have been a key driver in the much more recent evolution of pathogens such as the Gram positive bacterium *Staphylococcus aureus*.^9^–^11^ Methicillin resistant *Staphylococcus aureus* (MRSA), responsible for substantial morbidity and mortality worldwide, is one of the World Health Organisation's priority pathogens that represent a major threat to human health and which urgently require new therapeutic clinical options. Interestingly, although clinical use of the antibiotic drug methicillin has undoubtedly played a role in the spread of MRSA, it has recently been shown that acquisition of the methicillin resistance gene, *mecA*, by *S. aureus* probably occurred prior to the clinical introduction of methicillin,^12^ suggesting that other selection pressures may have driven the original emergence of MRSA. Whatever its origins, the factors that have facilitated the spread of MRSA, first as a hospital acquired infection that affected predominantly patients with weakened immune systems, but more recently as a community acquired disease able to infect otherwise healthy individuals, are of great interest.

The *mecA* gene, possession of which is the defining property of all MRSA isolates, is carried on mobile genetic elements that differ between MRSA lineages. It has recently been shown that genes encoding copper detoxification proteins are common to these mobile elements in distinct lineages of strain USA300,^9^ the current epidemic *S. aureus* lineage. These genes enable them to resist not only copper toxicity *in vitro*, but also copper-dependent macrophage killing.^10^,^11^ This suggests that these novel copper resistance genes, and copper tolerance more generally, may have been instrumental in the evolution of distinct lineages of MRSA.^9^–^11^ Importantly, copper has been shown to accumulate at sites of infection in mammals^13^ and is utilised by macrophages to kill engulfed bacteria.^14^ Furthermore, the virulence of numerous bacterial pathogens is ameliorated by mutations that reduce their ability to detoxify excess copper.^15^–^18^ Transcriptional induction of copper detoxification systems was observed in an MRSA strain cultured in serum or in blood.^19^ Taken together, these observations suggest that the acquisition of additional copper detoxification systems may have been a key driver in the evolution and spread of the current MRSA epidemic strains.

There is current interest in the application of the antimicrobial properties of copper in the clinic, for example by using copper alloys in touch surfaces such as door handles in hospitals to reduce the spread of nosocomial infections caused by pathogens such as MRSA.^20^–^22^ Yet the cellular mechanisms by which *S. aureus* handles copper, or how it responds to copper toxicity, have not been extensively studied to date. Here, we have used quantitative analysis of the *S. aureus* proteome under copper stress growth conditions to assess how this bacterium adapts to high levels of exogenous copper, finding that copper affects bacterial metal homeostasis and central carbon metabolism, as well as inducing oxidative and cell envelope stress responses. We then used metalloproteomic methods to determine intracellular metal distribution. We identified an abundant cytosolic protein that acquires copper under such excess copper growth conditions, which is involved in central carbon metabolism. We conclude that the adaptation of this bacterium to elevated copper involves alteration of its central metabolism, which may be caused by inhibition of a key glycolytic enzyme, whereas the oxidative stress response plays only a minor role in adaptation to copper stress.

## Methods

### Bacterial growth

The strain of *Staphylococcus aureus* used throughout was SH1000,^23^ a derivative of NCTC 8325. Four types of media were used: lysogeny broth (LB) or tryptic soy broth (TSB) were used as rich media, whereas a modified Tris-buffered minimal (TM) medium^24^ (either with or without addition of 0.25% w/v glucose) or a chemically defined minimal (CDM) medium^25^ were used for more restrictive and controlled growth conditions.

For the growth assays, strains were inoculated into 10 mL of each growth medium and incubated for 16 h at 37 °C with 180 rpm orbital shaking. These overnight cultures were used to inoculate fresh media to an OD_595nm_ ∼ 0.03, which was split into 10 mL aliquots and supplemented with varying concentrations of CuSO_4_. The cultures were incubated at 37 °C, and OD_595nm_ readings obtained using a plate reader (Biotek ELX800UV).

### Preparation of samples for quantitative mass spectrometry analysis

*S. aureus* SH1000 were cultured in 10 mL media (TSB or TM) for 16 h at 37 °C. These cultures were used to inoculate fresh media to an OD_595nm_ ∼ 0.03, which was split into multiple 10 mL aliquots and supplemented with varying concentrations of CuSO_4_. The cultures were incubated for 6 h at 37 °C with 180 rpm orbital shaking, before the cells were pelleted (3005*g*, 10 min, 4 °C) and washed once with 20 mM Tris, pH 7.5, 10 mM EDTA, and then washed twice with 20 mM Tris, pH 7.5. Washed cells were resuspended in 250 μL of 20 mM Tris, pH 7.5, 150 mM NaCl and 0.1 g of glass beads were added, before cells were lysed using a bead beater (Biospec). The lysed cells were pelleted (10 000*g*, 10 min, 4 °C), then this centrifugation was repeated to ensure removal of the beads. The total protein concentration was determined *via* Bradford assay (Thermo Coomassie Protein Plus), and the samples were adjusted with the same buffer to a final protein concentration of 0.2 mg mL^–1^. A 90 μL aliquot of each sample was transferred to a deep 96-well plate, to which 96 μL of 100 mM ammonium bicarbonate, 10 μL acetonitrile, and 15 μL of 10 mM DTT were added. Samples were incubated at 70 °C for 30 min then allowed to cool to room temperature. Trypsin (10 μL of 30 ng mL^–1^ – Promega) was added to each sample, before the plate was sealed with film and incubated at 37 °C with 180 rpm shaking overnight, before adding 10 μL of 10% formic acid. The plate was briefly spun (22.7*g*, 2 min, 4 °C) and flash frozen over liquid nitrogen. The samples were stored at –80 °C.

### Quantitative proteomic analysis by LC-MS/MS

Trypsin digested whole cell samples were analysed using a Michrom Advance high performance liquid chromatography (HPLC) system with reversed phase chromatography coupled to a Thermo Scientific Fusion hybrid Orbitrap ion trap mass spectrometer with a Michrom Advance CaptiveSpray source. Each sample was concentrated onto a trap column (0.2 × 10 mm ID, 5 μm particle size, 120 Å pore size, C18 Reprosil-Gold – Dr Maisch GmbH) and rinsed with 100 μL 0.1% formic acid, 2% acetonitrile, 97.9% water before gradient elution through a reversed phase C18 column (0.1 × 400 mm ID, 3 μm particle size, 120 Å pore size, C18 Reprosil-Gold, Dr Maisch GmbH) at a flow rate of 300 nL min^–1^. The chromatography consisted of a nonlinear 220 min gradient from 5% to 95% buffer B, where buffer A was 0.1% formic acid in water and buffer B was 0.1% formic acid in acetonitrile (all solvents were Fisher Optima grade). The mass spectrometer monitored MS1 scans from 380 *m*/*z* to 1580 *m*/*z* at 240 K resolution. MS2 scans were performed using the ion trap on the top N ions with an isolation window of 1.4 *m*/*z* and a 15 second exclusion time.

Mass spectra were searched against the *Staphylococcus aureus* NCTC8325 translated transcriptome using Proteome Discoverer 1.4 using the SEQUEST HT algorithm (Thermo) with a parent tolerance of 10 ppm and a fragment tolerance of 0.6 Da for the Fusion. Proteome Discoverer output files were then loaded in Scaffold (Proteome Software) with a protein threshold maximum of 1.0% false discovery rate.

### Elemental analysis by ICP-MS

For analysis of chromatographic fractions, aliquots were diluted (5–50-fold, depending on protein concentration and salt content) in 2.5% nitric acid containing 20 μg L^–1^ platinum and silver as internal standards, and analysed for total metal content using inductively coupled plasma mass spectrometry (ICP-MS; Thermo x-series). Each sample was analysed (100 reads, 30 ms dwell, 3 channels, 0.02 atomic mass unit separation, in triplicate) for ^55^Mn, ^65^Cu, ^66^Zn, ^107^Ag, and ^195^Pt in standard mode, and metal concentrations determined by comparison to matrix-matched elemental standard solutions.

For analysis of whole cells, *S. aureus* were cultured in 10 mL TM for 16 h at 37 °C. These cultures were used to inoculate fresh TM medium, either with or without the addition of 1 mM CuSO_4_, to an OD_595nm_ ∼ 0.03. After 6 h incubation (37 °C with 180 rpm orbital shaking), the final OD_595nm_ readings were taken, and equal numbers of the cells were harvested by centrifugation (3005*g*, 10 min, 4 °C), washed with 20 mM Tris, pH 7.5, 10 mM EDTA, and then washed twice with 20 mM Tris, pH 7.5. Washed pellets were digested with 65% (w/v) nitric acid (Merck) for 48 h at room temperature. Digested cells were then diluted 20-fold in 2.5% nitric acid containing 20 μg L^–1^ platinum and silver as internal standards, and analysed by ICP-MS in collision cell mode (using 3 mL min^–1^ 8% H_2_ in He as collision gas). Acid digested cell samples were analysed (100 reads, 30 ms dwell, 5 channels, 0.02 atomic mass unit separation, in triplicate) for ^55^Mn, ^65^Cu, ^66^Zn, ^107^Ag, and ^195^Pt. Metal concentrations were determined by comparison to matrix-matched elemental standard solutions, and normalised to account for small differences in the final OD readings.

### Metalloproteomic separation of *S. aureus* extracts

*S. aureus* were cultured in TSB, with or without supplementation with either 50 μM (Fig. 4) or 500 μM (Fig. 3) CuSO_4_, in cultures of 0.5 L in large baffled flasks (2.5 L total volume) at 37 °C with constant 180 rpm orbital shaking. For the comparative analysis of the wild type (WT) and Δ*gapA* mutant strains (Fig. 5), cells were cultured in TM medium lacking glucose, with or without supplementation with 50 μM CuSO_4_, under the same growth conditions. For the small-scale analysis comparing the WT with the Δ*sodA*Δ*sodM* mutant strain, cultures were prepared at 100 mL volume of TSB. Cells were harvested (4000*g*, 30 min, 4 °C) and washed once with 100 mL of 10 mM Tris, pH 7.5, 10 mM EDTA, followed by two washes with 10 mM Tris, pH 7.5, and stored at –80 °C.

Biomass was thawed on ice and resuspended in buffer and lysed by freeze grinding in liquid nitrogen. The frozen lysate was immediately transferred to an N_2_ glove-box (Belle Technologies, UK) and, once thawed, the cell lysate was sealed anaerobically and centrifuged (3005*g*, 20 min, 4 °C). The supernatant, sealed anaerobically, was further ultra-centrifuged (159 641*g*, 30 min, 4 °C) to remove insoluble material.

For the data presented in Fig. 4, the crude soluble lysate was lysed in 5 mM sodium phosphate, pH 6.8, and first applied to a 5 mL ceramic hydroxyapatite (CHT) column (Bio-Rad) on an AKTA fast performance liquid chromatography (FPLC) system (GE Healthcare). The flow-through from this column retained all of the copper content of the sample (data not shown), so this was concentrated using a 10 kDa cut-off concentrator (Amicon), and then diluted 70-fold in 15 mL of 10 mM Tris, pH 8.0 for subsequent chromatography.

The hydroxyapatite column flow-through for the analysis shown in Fig. 4, or in all other cases the crude soluble lysate (Fig. 3, 5 and Fig. S4, S5, ESI†), was loaded on a 1 mL HiTrap Q HP anion exchange chromatography (AEC) column (GE Healthcare) in 20 mM Hepes, pH 8.0 inside the anaerobic chamber (Belle Technology). This was eluted either (a) with a linear NaCl gradient over 40 column volumes using degassed buffers (Buffer A: 20 mM Hepes, pH 8.0; Buffer B: 20 mM Hepes, pH 8.0, 1 M NaCl) on an AKTA FPLC system (Fig. 4), or (b) with stepwise elution with increasing (100, 200, 300, 400, 500, and 1000 mM) NaCl concentration in 20 mM Hepes, pH 8.0, inside the anaerobic chamber (Fig. 3, 5 and Fig. S4, S5, ESI†).

The fractions eluted from AEC were subsequently resolved by a further dimension of size exclusion chromatography (SEC). This was either (a) a Superdex 200 Increase (10/300, GE Healthcare), resolved in 20 mM Tris, pH 7.5, 150 mM NaCl, using degassed buffers on the AKTA FPLC (Fig. 4 and 5), or (b) SW3000 column (Tosoh Biosciences) in 5 mM Hepes, pH 7.5, 50 mM NaCl (Fig. 3 and Fig. S2–S5, ESI†).

Fractions collected from SEC were analysed for metal content by ICP-MS, and for protein by *A*_280nm_, Bradford assay, or by SDS-PAGE.

### Electrophoretic analyses

Protein in SEC fractions were visualised by SDS-PAGE through staining with Sypro Ruby (Thermo Fisher) or Oriole (Bio-Rad) fluorescent stain, scanned on a Chemidoc (BioRad), and analysed using ImageJ.^26^,^27^

DNA samples were analysed using agarose gel electrophoresis on 1% (w/v) agarose gels in TAE buffer (40 mM Tris, 20 mM acetic acid, and 1 mM EDTA, pH 8.0) containing 0.01% SYBR® Safe DNA Gel Stain (Thermo-Fisher), and visualised using the ChemiDoc.

The superoxide dismutase activity was assessed using the in-gel nitro blue tetrazolium chloride (NBT – Sigma-Aldrich, UK) negative staining assay.^28^ The SEC eluate fractions were resolved on non-denaturing conditions 12% polyacrylamide gels and stained with 0.5 mM NBT, 28 mM TEMED, 28 μM riboflavin in 100 mM sodium phosphate pH 7.0 buffer. The gel was incubated in 20 mL of NBT-riboflavin stain at room temperature in the dark for 20 min, and then visualised by exposing the gel to bright white light. The gels were scanned using a Bio-Rad ChemiDoc system with a white light conversion screen.

### Identification of proteins in chromatographic fractions

Target protein bands were cut from acrylamide gels, trypsin-digested and analysed on a Voyager-DE (ABI) matrix-assisted laser desorption ionization time-of-flight (MALDI-TOF) mass spectrometer, as previously described.^26^,^27^,^29^ Peptide mass fingerprints were analysed using the Mascot search tool (; matrixscience.com).

### Molecular cloning and phage transduction

The *gapA* gene, including its Shine–Dalgarno sequence, was amplified from *S. aureus* SH1000 genomic DNA with primers gapA-amp-F (5′-GGGGGATCCAGGAGGCCATTATAATGGCAGTAAAAG) and gapA-amp-R (5′-GGGCTGCAGTTATTTAGAAAGTTCAGCTAAG), and ligated into the pS10t plasmid^30^ using *Bam*HI and *Pst*I restriction cloning. The ligations were transformed into *E. coli* DH5α cells and the identity of a positive clone was confirmed by Sanger sequencing with M13 primers (GATC). The pS10-gapA plasmid was transformed into *S. aureus* RN4220 and subsequently into *S. aureus* SH1000.

For phage transduction, the donor strain (*S. aureus* 8325-4 Δ*gapA*^31^) was incubated in 5 mL LB overnight and then sub-cultured into 25 mL fresh LB containing 10 mM CaCl_2_, inoculating to an OD_600nm_ ∼ 0.05. Strains were incubated at 37 °C until OD_600nm_ ∼ 0.2 was reached. A 10 mL aliquot of this culture was diluted with 25 mL fresh LB containing 10 mM CaCl_2_, and 1 mL of phage φ11 (a kind gift from Dr Julie Morrissey, University of Leicester) was added. This mixture was incubated at 37 °C until complete lysis had occurred (∼4 h). Cell debris was pelleted (4000*g*, 15 min, 4 °C) and the lysate sterilised through a 0.25 μm filter. The sterilised lysate was stored at 4 °C until required.

The recipient strain, SH1000, was incubated in 20 mL TM broth overnight, pelleted (4000*g*, 10 min, room temperature) and resuspended in 1 mL of fresh TM broth. An aliquot (0.5 mL) of cells was combined with 1 mL TM containing 10 mM CaCl_2_ and 0.5 mL donor phage φ11. The other aliquot (0.5 mL) of cells was used in a negative control containing no phage. Samples were incubated at 37 °C statically for 25 min, and then with shaking for 15 min. A 1 mL aliquot of ice-cold 20 mM sodium citrate was added, and the cells pelleted (4000*g*, 10 min, 4 °C), before being resuspended in 1 mL 20 mM sodium citrate and incubated on ice for 2 h. Transduction samples were plated onto TM agar containing selective 10 μg mL^–1^ tetracycline and 0.05% sodium citrate, and incubated at 37 °C.

Genomic DNA was prepared from transductants and screened by PCR using primers Type-gapA-F (5′-GGTAGAATTGGTCGTTTAGC) and Type-gapA-R (5′-GAAAGTTCAGCTAAGTATGC), which demonstrated insertion of the antibiotic resistance cassette. To verify successful transduction, the *rsbU* gene, which differs between *S. aureus* 8325-4 and SH1000 strains,^23^ was amplified by PCR from genomic DNA (Type-rsbU-F 5′-TCAAATTATTATATACCCATC and Type-rsbU-R 5′-CCTTGCTTAAGCATTTGC), yielding an ∼1 kb PCR product, and the wild type *rsbU* status of the SH1000 Δ*gapA* transductant strain confirmed by a diagnostic restriction digest of this product with *Apo*I enzyme (NEB).

### Glyceraldehyde-3-phosphate dehydrogenase activity assay

The glyceraldehyde-3-phosphate dehydrogenase (GAPDH) assay was performed in an assay mix, initiated when 100 μL aliquots of 100 mM NaHPO_4_, pH 7.5, 10 mM EDTA, 80 mM triethanolamine, 8 mM glyceraldehyde-3-phosphate (Sigma), 4 mM nicotinamide adenine dinucleotide (NAD^+^ – Sigma-Aldrich) was added to 100 μL samples of anaerobically prepared SEC eluate or of diluted whole cell soluble lysate (50 μg protein in 100 μL) in a flat-bottomed 96-well plate. The reaction was followed by measuring *A*_340nm_ every 20 s for 20 min on a Biotek ELX800UV plate reader. Negative controls (lacking either G3P, NAD^+^, or protein) were assayed alongside the samples, as were positive controls using commercial GAPDH enzyme (Sigma). Where required, either Cu(i) (prepared as previously described^29^), Ni(ii), Zn(ii), Co(ii), or bathocuproine disulfonate (BCS) were added anaerobically to the samples and incubated for 10 min, before addition of the assay buffer.

### Preparation of RNA and quantitative analysis of transcripts

*S. aureus* strains were cultured in TM for 5 h (from a starting OD_595nm_ of 0.03) before being shocked by addition of 2 mM CuSO_4_ and incubated for a further 20 min. Cells were spun down, resuspended in RNAlater (Ambion) and incubated at 4 °C overnight. RNA was extracted using the total RNA extraction kit (Norgen) according to the manufacturer's protocol with the following additions; to lyse cells, lysostaphin (0.4 mg mL^–1^) was added to the TE buffer and incubated at 37 °C for 30 min, and β-mercaptoethanol (1% v/v) was added to the RL buffer before use. To remove any remaining genomic DNA, the RNA was treated with TURBO DNase (Invitrogen) following manufacturer's instructions. A total of 2 μg of RNA was converted to cDNA using the SuperScript VILO cDNA synthesis kit (Invitrogen) and 50 ng used per qPCR reaction. The qPCR reactions were conducted using SYBR Green I Master mix (Roche) in a LightCycler 480 (Roche), using primer pairs for *copA* (primer Q-copA-F 5′-CGGTGTAAATGATGCACCTG-3′ and primer Q-copA-R 5′-TGCTTCAATGGCAACTTCTG-3′), *gapA* (primer Q-gapA-F 5′-TGCAAGGTCGTTTCACAGGT-3′ and primer Q-gapA-R 5′-TGCTTGCATCTGGTTCACTGA-3′). Relative gene expression was determined using the ΔΔCt method to calculate RQ (2-ΔΔCt). Expression was normalised to that of the endogenous control gene, *gyrB* (using primer Q-gyrB-F 5′-GACTGATGCCGATGTGGA-3′ and Q-gyrB-R 5′-AACGGTGGCTGTGCAATA-3′), and calibrated against SH1000 cultured without exogenous copper. All results are averages of 3 independent experiments, with technical triplicates being conducted for each qPCR experiment.

## Results

### Growth of *S. aureus* is inhibited by excess exogenous copper

**Fig. 1:**
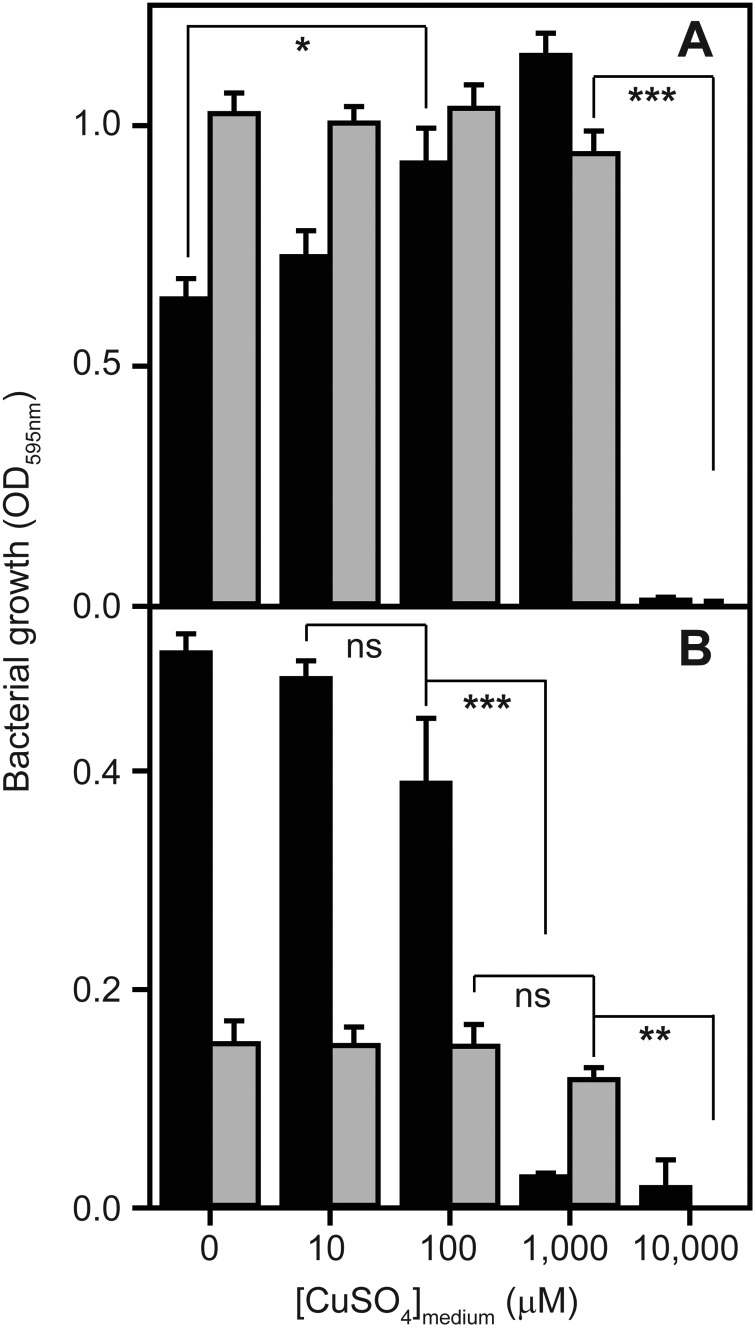
High concentrations of copper inhibit the growth of *S. aureus*. Wild type *S. aureus* SH1000 were inoculated to a starting OD_595nm_ of 0.03 into (A) two types of rich media, tryptic soy broth (TSB – black bars) or LB (grey bars), or (B) two types of minimal media, glucose-containing chemically defined medium (CDM – black bars) or Tris-minimal (TM) medium lacking glucose (grey bars), each supplemented with increasing concentrations of CuSO_4_. The cultures were incubated for 5 h at 37 °C with orbital shaking (180 rpm), and the final OD_595nm_ values were recorded. Growth is inhibited by high levels of copper in all media, but this toxicity effect is greatest in the minimal media. The symbols represent statistical significance based on a Student's *t*-test, where ns = not significant, * = *p* < 0.05, ** = *p* < 0.005, and *** = *p* < 0.0005.

To determine to what extent high exogenous levels of copper affect the growth of *S. aureus*, we inoculated four different types of growth medium, each containing varying concentrations of CuSO_4_, with cells of the *S. aureus* strain SH1000 and monitored changes in culture density over time (Fig. 1). Although *S. aureus* exhibits significant inherent tolerance of growth in high copper, we observed that proliferation could be inhibited in all four growth media by high levels of exogenous copper. As has been reported elsewhere,^2^,^32^,^33^ the toxicity of copper was strongly affected by the composition of the growth medium. In rich media, growth was uninhibited by doses of up to 1 mM copper, but was completely inhibited by 10 mM (Fig. 1A). Intriguingly, growth was actually reproducibly stimulated by copper addition up to 1 mM in TSB (Fig. 1A), an effect similar to that previously reported for another *S. aureus* strain.^32^ The reasons for this stimulation are unclear, but it seems likely that it relates to displacement of other essential metal ions from metal-chelating ligands present in the medium, thereby increasing their bioavailability to bacterial uptake transporters, rather than to an elevated copper demand of *S. aureus*. Conversely, the growth of *S. aureus* SH1000 in a chemically defined minimal medium was found to be much more sensitive to copper toxicity, with growth inhibition observed at copper concentrations ≥1 mM (Fig. 1B). An alternative Tris-buffered minimal medium (TM), here lacking glucose, showed slower bacterial proliferation and significant growth inhibition only at concentrations >1 mM CuSO_4_ (Fig. 1B). This is consistent with a model in which the additional chemical complexity of rich media leads to more effective chelation of the copper ions by media components,^2^,^33^ thereby reducing the toxic effects of metal excess.

### Quantitative proteomic analysis reveals adaptive changes to high copper in *S. aureus*

To glean insight into what aspects of *S. aureus* physiology are affected by excess exogenous copper concentrations, and how *S. aureus* adapts in order to be able to grow in the presence of copper excess, we used quantitative proteomic analysis of *S. aureus* cell lysates to identify proteins whose abundances were altered during growth in media containing high copper. We prepared whole cell extracts for mass spectrometric analysis from *S. aureus* SH1000 cells cultured for 6 h in TM (in this case containing glucose to ensure robust growth) with or without either 1.0 or 1.5 mM CuSO_4_, and also from cells cultured in TSB with or without either 1.0 or 2.5 mM CuSO_4_. These doses were chosen to represent non-inhibitory and mildly inhibitory conditions, respectively, and to make the extent of growth inhibition similar between the two different growth media (for the proteomic samples: in TM, 1 mM Cu resulted in 88% of growth relative to control, and 1.5 mM Cu resulted in 68% of growth relative to control; in TSB, 1 mM Cu resulted in 93% of growth relative to control, and 2.5 mM Cu resulted in 73% of growth relative to control – data not shown). Whole cell extracts were prepared, and peptides resulting from trypsin digestion were separated by liquid chromatography and quantified by mass spectrometry.

The complete list of proteins whose abundances were significantly altered under these copper conditions in TSB are shown in Tables 1 and 2, and those altered under elevated copper conditions in TM are shown in Tables 3 and 4, whereas Tables 5 and 6 illustrate the proteins whose abundances were altered by both doses of copper in TSB and TM medium, respectively. The number of proteins whose abundances changed during growth in the presence of copper were surprisingly limited, suggesting that change in expression of a relatively small number of genes is necessary and sufficient to adapt to the main effects of elevated copper in each medium. Yet the proteins whose abundances were altered also differed substantially between cells in TM and those in TSB (compare Tables 1 and 2 to Tables 3 and 4 and Table 5 to Table 6), implying that the copper-adaptive responses of *S. aureus* to high copper, just like toxicity, are highly dependent on the composition of the growth medium.

**Table 1:** All proteins whose abundances were significantly changed after growth for 6 h in TSB medium in the presence of 1.0 mM CuSO_4_, relative to control cells in TSB alone, as determined by LC-MS/MS. Column ‘*P*’ shows statistical significance using the Fisher's exact test

Protein	Gene	ORF	GI	Fold	*P*
*Proteins with increased abundance in cells cultured in 1 mM Cu in TSB medium*					
Copper-exporting P-type ATPase	*copA*	SAOUHSC_02873	gi|122538628	ON	<0.00010
30S ribosomal protein S12	*rpsL*	SAOUHSC_00527	gi|447065081	10	<0.00010
Copper chaperone CopZ	*copZ*	SAOUHSC_02874	gi|122538627	7.3	<0.00010
Cysteine synthase	*cysK*	SAOUHSC_00488	gi|445979739	2.3	<0.00010
Fructose-bisphosphate aldolase	*fda*	SAOUHSC_02926	gi|446954157	2.3	<0.00010
Phosphoribosylformylglycinamidine synthase	*purS*	SAOUHSC_01011	gi|446771094	1.9	0.00012
Pyruvate dehydrogenase E1 component subunit beta	*pdhB*	SAOUHSC_01041	gi|445990321	1.6	<0.00010
Glyceraldehyde-3-phosphate dehydrogenase A	*gapA*	SAOUHSC_00795	gi|446201559	1.4	<0.00010

*Proteins with decreased abundance in cells cultured in 1 mM Cu in TSB medium*					
Formate acetyltransferase	*pflB*	SAOUHSC_00187	gi|446817404	0.7	<0.00010
Putative formate dehydrogenase subunit alpha	*fdhA*	SAOUHSC_02582	gi|123406992	0.4	0.00041
Phenol-soluble modulin alpha 1 peptide	*psmA1*	SAOUHSC_00411.4	gi|206557863	0.2	<0.00010
Beta-class phenol-soluble modulin	*psmB2*	SAOUHSC_01136	gi|446320817	0.2	<0.00010
Phenol-soluble modulin alpha 2 peptide	*psmA2*	SAOUHSC_00411.3	gi|206557875	0.1	<0.00010
Serine-protein kinase, anti-sigma-B factor	*rsbW*	SAOUHSC_002299	gi|114152144	OFF	<0.00010

**Table 2:** All proteins whose abundances were significantly changed after growth for 6 h in TSB medium in the presence of 2.5 mM CuSO_4_, relative to control cells in TSB alone, as determined by LC-MS/MS. Column ‘*P*’ shows statistical significance using the Fisher's exact test

Protein	Gene	ORF	GI	Fold	*P*
*Proteins with increased abundance in cells cultured in 2.5 mM Cu in TSB medium*					
Copper-exporting P-type ATPase	*copA*	SAOUHSC_02873	gi|122538628	ON	<0.00010
30S ribosomal protein S15	*rpsO*	SAOUHSC_01250	gi|118597468	12	<0.00010
30S ribosomal protein S12	*rpsL*	SAOUHSC_00527	gi|447065081	9	<0.00010
Copper chaperone CopZ	*copZ*	SAOUHSC_02874	gi|122538627	6.9	<0.00010
Superoxide dismutase [Mn/Fe] 2	*sodM*	SAOUHSC_00093	gi|123003520	6.7	0.00025
Virulence factor EsxA	*esxA*	SAOUHSC_00257	gi|447163570	3.1	<0.00010
Enoyl-[acyl-carrier-protein] reductase [NADPH] FabI	*fabI*	SAOUHSC_00947	gi|122539868	3.1	<0.00010
Alkyl hydroperoxide reductase subunit C	*ahpC*	SAOUHSC_00365	gi|60391219	2.2	<0.00010
Fructose-bisphosphate aldolase	*fda*	SAOUHSC_02926	gi|446954157	2.2	<0.00010
Transketolase	*tkt*	SAOUHSC_01337	gi|446403588	2	<0.00010
Alkyl hydroperoxide reductase subunit F	*ahpF*	SAOUHSC_00364	gi|3913011	1.8	<0.00010
Cysteine synthase	*cysK*	SAOUHSC_00488	gi|445979739	1.7	<0.00010
Hypothetical protein	—	SAOUHSC_00906	gi|122539893	1.7	0.00078
Glyceraldehyde-3-phosphate dehydrogenase A	*gapA*	SAOUHSC_00795	gi|446201559	1.3	<0.00010

*Proteins with decreased abundance in cells cultured in 2.5 mM Cu in TSB medium*					
Elongation factor Tu	*tuf*	SAOUHSC_00530	gi|123098060	0.8	<0.00010
Elongation factor G	*fusA*	SAOUHSC_00529	gi|119368769	0.7	<0.00010
DNA-directed RNA polymerase subunit beta′	*rpoC*	SAOUHSC_00525	gi|114152142	0.7	0.00032
Bifunctional autolysin	*atl*	SAOUHSC_00994	gi|110832765	0.7	<0.00010
Formate-tetrahydrofolate ligase	*fhs*	SAOUHSC_01845	gi|122540563	0.7	0.00025
Cold-shock protein [bacteria]	*cspC*	SAOUHSC_00819	gi|446981826	0.5	0.0001
NADH dehydrogenase-like protein	*ndh2*	SAOUHSC_00878	gi|122539909	0.5	0.00012
Adenylosuccinate synthetase	*purA*	SAOUHSC_00019	gi|446017472	0.4	0.00011
Putative formate dehydrogenase subunit alpha	*fdhA*	SAOUHSC_02582	gi|123406992	0.4	0.00046
Alcohol dehydrogenase	*adhA*	SAOUHSC_00608	gi|122540071	0.3	<0.00010
Formate acetyltransferase	*pflB*	SAOUHSC_00187	gi|446817404	0.1	<0.00010
Bifunctional acetaldehyde-CoA/alcohol dehydrogenase	*adhE*	SAOUHSC_00113	gi|88193926	0.1	<0.00010
Leukocidin-like protein 2	*lukH*	SAOUHSC_02243	gi|122539025	0.1	<0.00010
Leukocidin-like protein 1	*lukG*	SAOUHSC_02241	gi|122539026	0.06	0.00014
Anaerobic ribonucleoside triphosphate reductase	*nrdD*	SAOUHSC_02942	gi|88196568	0.04	<0.00010
Phenol-soluble modulin alpha 1 peptide	*psmA1*	SAOUHSC_00411.4	gi|206557863	0.04	<0.00010
Assimilatory nitrite reductase [NAD(P)H] large subunit	*nasD*	SAOUHSC_02684	gi|88196324	0.03	<0.00010
Beta-class phenol-soluble modulin	*psmB2*	SAOUHSC_01135	gi|446320817	0.03	<0.00010
Hypothetical protein	—	SAOUHSC_00144	gi|88193956	OFF	<0.00010
Mevalonate kinase	*mvaK1*	SAOUHSC_00577	gi|446119179	OFF	0.00097
Phenol-soluble modulin alpha 2 peptide	*psmA2*	SAOUHSC_00411.3	gi|206557875	OFF	<0.00010

**Table 3:** All proteins whose abundances were significantly changed after growth for 6 h in TM medium in the presence of 1.0 mM CuSO_4_, relative to control cells in TM alone, as determined by LC-MS/MS. Column ‘*P*’ shows statistical significance using the Fisher's exact test

Protein	Gene	ORF	GI	Fold	*P*
*Proteins with increased abundance in cells cultured in 1 mM Cu in TM medium*					
Copper chaperone CopZ	*copZ*	SAOUHSC_02874	gi|122538627	10	<0.00010
30S ribosomal protein S19	*rpsS*	SAOUHSC_02508	gi|446046498	2.5	0.00013
2,3-Bisphosphoglycerate-dependent phosphoglycerate mutase	*gpmA*	SAOUHSC_02703	gi|122538743	2.1	<0.00010
d-Alanine-poly(phosphoribitol) ligase subunit 1	*dltA*	SAOUHSC_00869	gi|122539917	2	<0.00010
Pyruvate carboxylase	*pycA*	SAOUHSC_01064	gi|88194813	1.3	0.00013
Histone-like DNA-binding protein HU	*hup*	SAOUHSC_01490	gi|446966607	1.2	<0.00010

*Proteins with decreased abundance in cells cultured in 1 mM Cu in TM medium*					
Cold-shock protein	*cspC*	SAOUHSC_00819	gi|446981826	0.1	<0.00010
Formate acetyltransferase	*pflB*	SAOUHSC_00187	gi|446817404	0.1	0.00017
30S ribosomal protein S12	*rpsL*	SAOUHSC_00527	gi|447065081	OFF	<0.00010
Serine-protein kinase, anti-sigma-B factor	*rsbW*	SAOUHSC_02299	gi|114152144	OFF	<0.00010

**Table 4:** All proteins whose abundances were significantly changed after growth for 6 h in TM medium in the presence of 1.5 mM CuSO_4_, relative to control cells in TM alone, as determined by LC-MS/MS. Column ‘*P*’ shows statistical significance using the Fisher's exact test

Protein	Gene	ORF	GI	Fold	*P*
*Proteins with increased abundance in cells cultured in 1.5 mM Cu in TM medium*					
Copper chaperone CopZ	*copZ*	SAOUHSC_02874	gi|122538627	5.3	<0.00010
d-Alanine-poly(phosphoribitol) ligase subunit 1	*dltA*	SAOUHSC_00869	gi|122539917	1.4	<0.00010
50S ribosomal protein L29	*rpmC*	SAOUHSC_02504	gi|122538860	1.4	0.00017
2,3-Bisphosphoglycerate-dependent phosphoglycerate mutase	*gpmA*	SAUOHSC_02703	gi|122538743	1.1	<0.00010
Alkyl hydroperoxide reductase subunit C	*ahpC*	SAOUHSC_00365	gi|60391219	1.1	<0.00010
Catalase	*katA*	SAOUHSC_01327	gi|126215678	<1.1	<0.00010

*Proteins with decreased abundance in cells cultured in 1.5 mM Cu in TM medium*					
Fructose-bisphosphate aldolase	*fda*	SAOUHSC_02926	gi|446954157	0.9	<0.00010
Glutamine synthetase	*glnA*	SAOUHSC_01287	gi|447049347	0.9	<0.00010
Transketolase	*tkt*	SAOUHSC_01337	gi|446403588	0.8	<0.00010
Histone-like DNA-binding protein HU	*hup*	SAOUHSC_01490	gi|446966607	0.8	<0.00010
30S ribosomal protein S1	*rpsA*	SAOUHSC_01493	gi|88195210	0.8	<0.00010
Hypothetical protein, family UPF0342	—	SAOUHSC_01977	gi|122539212	0.8	<0.00010
30S ribosomal protein S7	*rpsG*	SAOUHSC_00528	gi|110282992	0.7	0.00015
Branched-chain alpha-keto acid dehydrogenase subunit E2	*pdhC*	SAOUHSC_01042	gi|88194794	0.6	<0.00010
Bifunctional autolysin	*atl*	SAOUHSC_00994	gi|110832765	0.6	0.00038
Pyruvate carboxylase	*pycA*	SAOUHSC_01064	gi|88194813	0.6	<0.00010
Phosphoribosylamine-glycine ligase	*purD*	SAOUHSC_01018	gi|88194772	0.6	0.00074
DNA-directed RNA polymerase subunit beta′	*rpoC*	SAOUHSC_00525	gi|114152142	0.5	0.00013
Elongation factor Tu	*tuf*	SAOUHSC_00530	gi|123098060	0.2	<0.00010
Alkaline shock protein 23	*asp23*	SAOUHSC_02441	gi|446137381	0.1	<0.00010
Cell division protein FtsZ	*ftsZ*	SAOUHSC_01150	gi|122539740	0.08	0.00052
l-Lactate dehydrogenase 1	*ldh*	SAOUHSC_00206	gi|205438378	OFF	<0.00010
50S ribosomal protein L23	*rplW*	SAOUHSC_02510	gi|123003430	OFF	<0.00010
Cold-shock protein	*cspC*	SAOUHSC_00819	gi|446981826	OFF	<0.00010
50S ribosomal protein L13	*rplM*	SAOUHSC_02478	gi|122538869	OFF	<0.00010
50S ribosomal protein L18	*rplR*	SAOUHSC_02495	gi|115504959	OFF	0.00012

**Table 5:** Consolidated dataset of all proteins whose abundances were significantly changed after exposure to both copper doses during growth in TSB, relative to control cells

Protein	Gene	ORF	Fold (1.0 mM)	Fold (2.5 mM)
Copper-exporting P-type ATPase	*copA*	SAOUHSC_02873	ON	ON
30S ribosomal protein S12	*rpsL*	SAOUHSC_00527	10	9
Copper chaperone CopZ	*copZ*	SAOUHSC_02874	7.3	6.9
Cysteine synthase	*cysK*	SAOUHSC_00488	2.3	1.7
Fructose-bisphosphate aldolase	*fda*	SAOUHSC_02926	2.3	2.2
Glyceraldehyde-3-phosphate dehydrogenase A	*gapA*	SAOUHSC_00795	1.4	1.3
Formate acetyltransferase	*pflB*	SAOUHSC_00187	0.7	0.1
Putative formate dehydrogenase subunit alpha	*fdhA*	SAOUHSC_02582	0.4	0.4
Phenol-soluble modulin alpha 1 peptide	*psmA1*	SAOUHSC_00411.4	0.2	0.04
Beta-class phenol-soluble modulin	*psmB2*	SAOUHSC_01136	0.2	0.03
Phenol-soluble modulin alpha 2 peptide	*psmA2*	SAOUHSC_00411.3	0.1	OFF

**Table 6:** Consolidated dataset of all proteins whose abundances were significantly changed after exposure to both copper doses during growth in TM, relative to control cells

Protein	Gene	ORF	Fold (1.0 mM)	Fold (2.5 mM)
Copper chaperone CopZ	*copZ*	SAOUHSC_02874	10	5.3
2,3-Bisphosphoglycerate-dependent phosphoglycerate mutase	*gpmA*	SAOUHSC_02703	2.1	1.1
d-Alanine-poly(phosphoribitol) ligase subunit 1	*dltA*	SAOUHSC_00869	2	1.4
Cold-shock protein	*cspC*	SAOUHSC_00819	0.1	OFF

### Induction of metal homeostasis systems

The most striking observation from the proteomic analysis was the strong induction of components of the known *S. aureus* copper detoxification system. This system consists of the *copAZ* operon, encoding a copper-exporting P-type ATPase CopA and associated copper metallochaperone CopZ, respectively, which are known to be induced in a copper-dependent manner by the copper-sensing transcriptional regulator CsoR.^10^,^32^,^34^,^35^ The CopA pump was not detected in either control sample, but was detected in both elevated Cu conditions in TSB medium (Tables 1 and 2), representing copper-dependent induction of the efflux pump. Interestingly, no peptides from CopA were detected in the extracts prepared under elevated copper conditions in TM medium (Tables 3 and 4) for reasons that are unclear. However, given that CopA is an integral membrane protein and our extraction protocol was not specifically designed to solubilise membrane proteins, the quantitation of CopA should be treated with some caution. Conversely, the soluble copper metallochaperone CopZ was detected in all 6 samples, and was induced from basal levels under control conditions in both media to be detected at elevated levels under both copper doses (CopZ was induced 7.3- and 6.9-fold in 1.0 and 2.5 mM Cu respectively in TSB medium relative to control, and induced 10- and 5.3-fold in 1.0 and 1.5 mM Cu respectively in TM medium relative to control – Tables 1–4). Together, these data demonstrate the importance of the known copper-specific regulatory network in *S. aureus* copper resistance, in which binding of copper to the copper sensor CsoR leads to de-repression of these two known components of copper homeostasis.

**Fig. 2:**
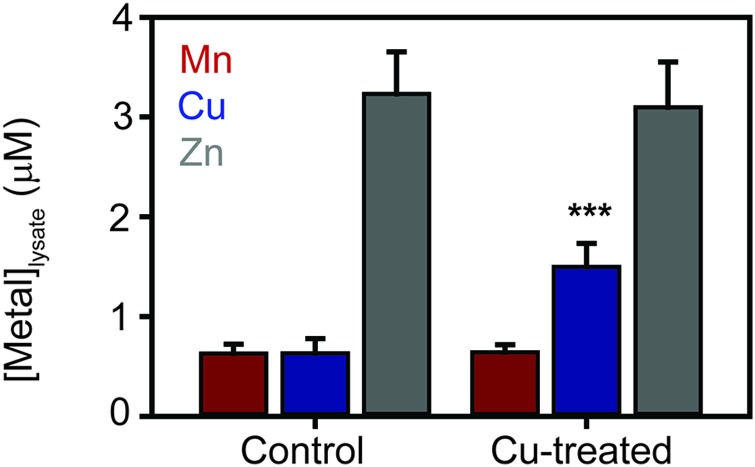
*S. aureus* cells accumulate additional copper when cultured in high concentrations of copper. Wild type *S. aureus* SH1000 were inoculated to a starting OD_595nm_ of 0.05 into TM media (containing glucose) either with (Cu-treated) or without (control) the addition of 1 mM CuSO_4_, and incubated for 6 h at 37 °C with 180 rpm orbital shaking. Cell densities were determined by OD_595nm_, and equal cell numbers were harvested, washed to remove surface-bound metals, digested in concentrated HNO_3_, and elemental composition of the resulting acid lysates was determined by ICP-MS. The symbols represent statistical significance, based on a Student's *t*-test, where *** = *p* < 0.0005.

No other known metal homeostasis proteins exhibited significant differential expression under these conditions. However, we noted that the inducible superoxide dismutase enzyme, SodM, was significantly induced 6.7-fold by 2.5 mM Cu in TSB relative to control (and was induced under all other copper treatments but failed to reach statistical significance). The *sodM* gene is known to be induced under manganese starvation conditions.^36^ A component of the high affinity manganese transporter, MntC, which is regulated by the manganese sensor MntR,^37^ also appeared to be induced under the copper treatment conditions, although this did not reach statistical significance (see full results table, provided as ESI†). These observations raised the possibility that copper-treated cells might experience some level of manganese starvation. To test this, we measured the metal content of *S. aureus* SH1000 cells after 6 h culture in TM medium containing 1 mM CuSO_4_ by inductively coupled plasma mass spectrometry (ICP-MS) relative to control cells cultured in the same medium without copper (Fig. 2). As expected, the cellular copper content of cells cultured in high copper conditions increased almost 2-fold, but no significant change in the abundance of cellular manganese or zinc content was observed (Fig. 2).

### Excess copper leads to induction of stress response and virulence proteins

A further observation from the proteomic analysis was the induction of proteins whose function is in oxidative stress defence. In the higher Cu dose (2.5 mM) in TSB medium, we detected induction of the aforementioned inducible SOD enzyme, SodM, as well as components of the alkyl hydroperoxide reductase (AhpC was induced 2.2-fold and AhpF was induced 1.8 fold under these conditions relative to control – Table 2). AhpC was also induced slightly (1.1-fold relative to control) during growth in TM medium in the presence of 1.5 mM Cu, as was the catalase enzyme KatA (Table 4). Notably, transcriptional induction by copper of the *sodM* and *ahpF* genes was previously described.^5^ Conversely, the cysteine synthase CysK was induced by both copper doses in TSB medium (2.3- and 1.7-fold in 1.0 and 2.5 mM Cu respectively relative to control – Tables 1 and 2), which may suggest thiol stress is caused to some extent by copper excess. There has been much controversy about the importance of direct copper-catalysed generation of reactive oxygen species (ROS) in the molecular mechanism of copper toxicity in bacteria.^2^,^4^ Notably, this *S. aureus* strain carries a mutation in the biosynthetic pathway for production of the low molecular weight thiol, bacillithiol,^38^ and no enzymes associated with the alternative, reactive sulfur species-mediated oxidative stress response^39^ were found to be induced by copper. We interpret the observed low-level induction of ROS defence enzymes, and the fact that this induction was observed only in the presence of the highest, but not of the lowest, doses of copper as signifying that ROS generation plays at most a minor role in copper toxicity in *S. aureus*.

Other cellular stresses were also evident from the proteomic analyses. All copper-treated cells showed differential expression of a number of ribosomal proteins, although in a complex manner (30S ribosomal proteins S12, S15, S19 and 50S ribosomal protein L29 were all increased in specific copper conditions, whereas 30S ribosomal proteins S1, S7, S12, and 50S ribosomal proteins L13 and L18 were decreased in other conditions – Tables 1–4). Notably, decreased abundance of elongation factor Tu (*tuf*; 0.8-fold during growth in the presence of 2.5 mM Cu in TSB, and 0.2-fold in the presence of 1.5 mM Cu in TM relative to control – Tables 2 and 4) and elongation factor G (*fusA*; 0.7-fold during growth in the presence of 2.5 mM Cu in TSB – Table 2) were also observed, implying copper stress impacts on ribosome structure and function. Genes encoding ribosomal proteins were previously shown to be transcriptionally down-regulated under copper stress.^5^ Transcription may also be affected by copper stress, as decreased abundance of the DNA-directed RNA polymerase subunit β′ that contains the polymerase active site was also observed during growth in the highest copper concentrations (*rpoC*; 0.7-fold during growth in the presence of 2.5 mM Cu in TSB, and 0.5-fold during growth in the presence of 1.5 mM Cu in TM relative to control – Tables 3 and 4), which also correlates with a previous transcriptional study.^5^ We conclude that copper excess results in complex stress effects on *S. aureus*, including alterations to transcription and, especially, on translation, but only limited oxidative stress.

Copper-dependent differences were also observed in the expression of a number of known virulence factors. The abundances of protein components of the phenol soluble modulins (encoded by the *psmA1*, *psmA2* and *psmB2* genes) were strongly reduced in both copper doses in TSB (Tables 1 and 2) but were unaffected by copper during growth in TM (Tables 3 and 4), whereas virulence factor EsxA was induced and the leukocidin-like proteins (encoded by the *lukG* and *lukH* genes) were simultaneously repressed during growth in the presence of 2.5 mM copper in TSB (Table 2). Interestingly, we also observed complete loss of serine-protein kinase RsbW in one sample (1.0 mM Cu) from each medium, which is the anti-sigma factor that regulates σ^B^ activity, which in turn regulates expression of diverse stress response and virulence genes.^40^ This regulatory activity of σ^B^ is essential for virulence and for intracellular survival within host cells.^41^,^42^ However, given that RsbW is predicted to contain a TM helix, this quantitation should be treated with some caution as the extraction protocol was not optimised to recover membrane proteins. Notably, we did not observe any significant down-regulation of expression of AgrD or SaeRS, which were previously shown to be transcriptionally down-regulated by copper,^5^ implying potential post-transcriptional control of the abundances of these proteins.

### Copper excess leads to changes in the *S. aureus* cell envelope

During growth under elevated copper conditions in the TM medium, we observed significantly increased expression of a subunit of the d-alanine-poly(phosphoribitol) ligase DltA (2.0- and 1.4-fold during growth in the presence of 1.0 and 1.5 mM Cu respectively in TM relative to control – Tables 3 and 4). This enzyme catalyses the first step in the d-alanylation of lipoteichoic acid (LTA) by loading the carrier protein DltC with d-alanine. Together these enzymes modify LTA, which modulates the chemical properties of the LTA and thus of the cell wall. In particular, LTA modification by the Dlt system reduces the negative charge on the cell wall, which in turn is known to alter the susceptibility of *S. aureus* cells to cationic antimicrobial peptides (CAMPs).^43^ This suggests that *S. aureus* adapts to copper stress by modifying the charge associated with its cell wall, presumably to reduce adventitious association of exogenous copper ions with the wall. We hypothesise that this cell wall modification in response to copper will result in increased tolerance of copper-treated *S. aureus* cells to CAMPs, although this hypothesis awaits experimental testing.

Some degree of cell wall stress induced by growth in elevated copper was also confirmed by the aforementioned abundance changes observed in the anti-sigma factor RsbW, which would be anticipated to increase transcription of σ^B^-dependent targets, and also by changes observed in the abundance of alkaline shock protein Asp23, itself a σ^B^-regulated protein.^40^,^44^ However, the cell wall stress response is difficult to interpret, as we observed strong down-regulation of Asp23 in the presence of copper in TM (0.1-fold during growth in the presence of 1.5 mM Cu in TM relative to control – Table 4), whereas increased Asp23 abundance might have been anticipated if this was mediated by changes in RsbW abundance. We also observed significant decreases in the abundance of a bifunctional autolysin (encoded by the *atl* gene) under the highest copper doses (0.7- and 0.6-fold induced during growth in the presence of 2.5 mM Cu in TSB and in the presence of 1.5 mM Cu in TM respectively, relative to their controls), which is proposed to be involved in controlled hydrolysis of peptidoglycan in the wall to allow growth and replication,^45^ also suggesting modulation of cell wall processes by excess exogenous copper. The observation of induction of the enoyl-(acyl carrier protein) reductase FabI (3.1-fold increased during growth in the presence of 2.5 mM Cu in TSB relative to control), which catalyses the rate-limiting step of fatty acid biosynthesis, also indicates potential cell envelope stress induced by excess copper (Table 2). Taken together, our proteomic analysis indicates that *S. aureus* adapts to copper stress by modifying the properties of the cell wall through the Dlt system and potentially also through modulation of cell wall stress responses.

### Excess copper affects *S. aureus* central carbon metabolism

Finally, a glaring implication of the proteomic analysis was that copper excess growth conditions resulted in adaptive changes to *S. aureus* central carbon metabolism. In TSB medium, two proteins of glycolysis were significantly induced. Fructose-bisphosphate aldolase (*fda*; 2.3- and 2.2-fold induced during growth in the presence of 1.0 and 2.5 mM Cu respectively in TSB medium relative to control) and the glyceraldehyde-3-phosphate dehydrogenase (GAPDH) glycolytic isozyme GapA^31^ (1.4- and 1.3-fold induced during growth in the presence of 1.0 and 2.5 mM Cu respectively in TSB medium relative to control) were induced (Tables 1 and 2), suggesting these adaptations are necessary during growth in the presence of copper to maintain adequate flux through central glycolysis. It's notable that, while the observed level of induction of GapA seems small, this enzyme was observed to be the most abundant protein detected in our proteomic analysis (see full results table, provided as ESI†); thus the energy investment made by the cell in even such modest induction is significant. These enzymes were not induced during growth in the presence of copper in TM medium (Tables 3 and 4). However, the 2,3-bisphosphoglycerate-dependent phosphoglycerate mutase enzyme GpmB was significantly induced by copper in TM medium (2.1- and 1.1-fold induced during growth in the presence of 1.0 and 1.5 mM Cu in TM medium relative to control – Tables 3 and 4). This enzyme catalyses the glycolytic or gluconeogenic interconversion of 3-phosphoglycerate and 2-phosphoglycerate, again suggesting a cellular response to deficient flux through glycolysis. It should be noted that negligible expression of the gluconeogenic GAPDH isozyme, GapB,^31^ was detected under all tested conditions (see full results table, provided as ESI†), consistent with provision of abundant glucose in all growth media for proteomic sampling.

Under these same conditions, we also observed altered expression of a number of enzymes involved in pyruvate metabolism: induction of the E1 component of the pyruvate dehydrogenase complex (*pdhB*: 1.6-fold induced during growth in the presence of 1.0 Cu in TSB medium relative to control – Table 1), which converts pyruvate to acetyl-CoA for entry into the tricarboxylic acid (TCA) cycle; repression of formate acetyltransferase (*pflB*: 0.7- and 0.1-fold during growth in the presence of 1.0 and 2.5 mM Cu respectively in TSB medium relative to control – Tables 1 and 2), which produces acetyl-CoA from pyruvate during anaerobic glucose metabolism; and repression of alcohol dehydrogenase (*adhA*: 0.4-fold during growth in the presence of 2.5 mM Cu in TSB relative to control – Table 2) and the bifunctional acetaldehyde-CoA/alcohol dehydrogenase (*adhE*: 0.1-fold during growth in the presence of 2.5 mM Cu in TSB relative to control – Table 2), which control the aerobic production of ethanol or acetate respectively, ultimately from pyruvate. In TM medium, we also observed changes in the abundance of formate acetyltransferase (*pflB*; 0.1-fold during growth in the presence of 1.0 mM Cu in TM relative to control – Table 3), as well as changes in abundance of further enzymes of pyruvate metabolism: changes in abundance of pyruvate carboxylase (*pycA*: 1.3- and 0.6-fold during growth in the presence of 1.0 and 1.5 mM Cu respectively in TM relative to control – Tables 3 and 4); and repression of l-lactate dehydrogenase (*ldh*: switched off during growth in the presence of 1.5 mM Cu in TM relative to control – Table 4), which catalyses lactate synthesis from pyruvate.

We conclude from these observations that copper excess results in altered central carbon metabolism in *S. aureus*. Specifically, *S. aureus* adaptations to excess exogenous copper involve the induction of several enzymes of glycolysis, including that which performs the rate-limiting step, GAPDH, while primarily down-regulating enzymes that metabolise pyruvate, the end-product of glycolysis. These metabolic changes enable *S. aureus* to continue to grow, albeit at a reduced rate, in the presence of high concentrations of copper.

### Cytosolic copper abundance is increased substantially under high copper growth conditions

The total cellular copper content of *S. aureus* was increased in cells exposed to excess exogenous copper relative to control cells (Fig. 2). We used an established metalloproteomic methodology to determine how this additional copper is distributed within the cell using anaerobic multi-dimensional native liquid chromatography to fractionate soluble extracts of *S. aureus* cells combined with ICP-MS elemental analysis.^26^,^27^,^29^,^46^–^48^

**Fig. 3:**
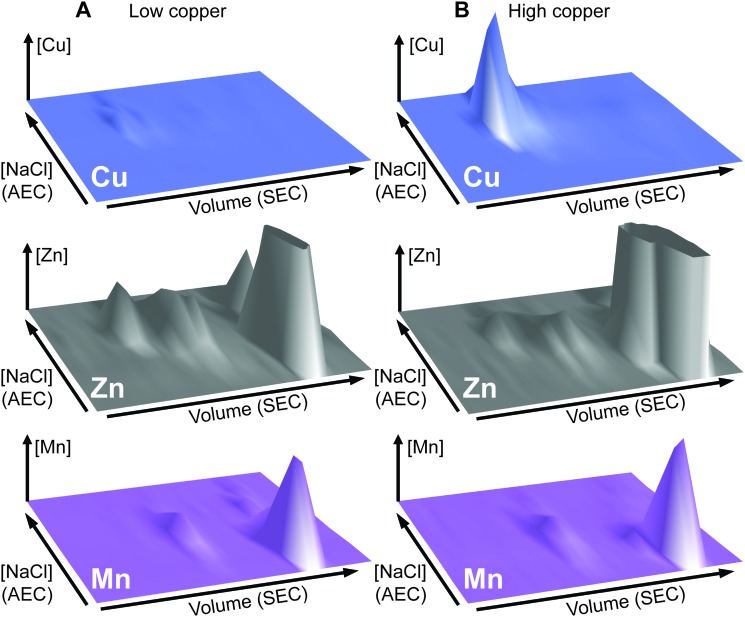
Metal distribution in *S. aureus* cytosolic extracts. Cytosolic extracts were prepared from *S. aureus* SH1000 cells, cultured in TSB medium either (A) without or (B) with addition of 500 μM CuSO_4_. Extracts were resolved by anion exchange chromatography (1 mL HiTrap Q HP column) in 20 mM Hepes, pH 8.0, and eluted with stepwise increases in NaCl concentration. Resulting fractions were subjected to a second dimension of separation by HPLC size exclusion chromatography (SW3000 column) in 5 mM Hepes, 50 mM NaCl, pH 7.5. Elemental composition of the resulting fractions were determined by ICP-MS, shown by the height of the peaks on the *z*-axis. Accumulation of copper in one major pool was observed in cells cultured in excess copper, whereas the accumulation of zinc was decreased in high molecular weight pools. Manganese accumulation was unchanged. All lysate preparation and chromatography steps were performed under anaerobic conditions.

We first prepared extracts from paired cultures of *S. aureus* SH1000; a control culture of cells grown for 6 h in TSB medium, and an otherwise identical culture of cells grown for 6 h in the same medium containing 0.5 mM CuSO_4_ throughout the culturing process. This concentration had no inhibitory effect on the growth of *S. aureus* (Fig. 1). Each extract was resolved identically by two dimensions of liquid chromatography; a first dimension of anion exchange chromatography (AEC), eluted with stepwise increases in NaCl concentration, followed by subjecting each fraction of AEC eluate to a second dimension of separation using size exclusion chromatography (SEC). To maintain native metal–protein associations, all extract preparation steps and the AEC were performed in an anaerobic glove box, and the SEC used thoroughly de-gassed and then nitrogen-saturated buffers. The resulting fractions were subjected to multi-elemental analysis by ICP-MS (Fig. 3).

This analysis, presented as two dimensional surface plots, demonstrated the presence of high molecular weight (MW), and thus presumably proteinaceous species, associated with manganese, zinc and copper (Fig. 3A). We also detected low MW pools of manganese and zinc, but not of copper, consistent with a model in which all copper is tightly associated with proteins.^49^ It should be noted that the high MW copper pools are not immediately obvious on the *z*-axis scale in which the data are shown in Fig. 3A, but they become very clear in an expanded view shown on an optimised *z*-axis (Fig. S1, ESI†). The identity of the proteins with which these pools of copper are associated in the extracts prepared from cells cultured in medium containing only basal levels of copper (ICP-MS analysis demonstrated the copper content of our TSB medium to be ∼0.13 μM), are currently unknown and are under investigation. They are particularly intriguing because the *S. aureus* SH1000 genome encodes no known soluble copper enzymes; the only anticipated copper proteins in the cytosol are the metallochaperone CopZ and the copper sensing transcriptional regulator CsoR,^32^,^34^,^35^ neither of which were detected in cells cultured in the absence of copper by quantitative proteomic analysis at levels that would suggest they could explain these data (ESI†). The equivalent metalloproteomic analysis of the extract prepared from cells cultured in medium containing excess copper (Fig. 3B) showed dramatic differences. The abundance of copper in the two dimensional surface plot was hugely increased, forming one large but poorly resolved high MW peak; yet still, no significant amount of low MW copper was observed (Fig. 3B).

A number of abundant zinc peaks were also detected in the lysates from the control cells (Fig. 3A), which were discrete from the copper peaks (Fig. S2, ESI†), suggesting the zinc is associated with different proteins from those with which the detected copper is associated. Interestingly, the hyper-accumulation of cytosolic copper in the copper-treated cells was accompanied by a reduction in the abundance of zinc in the high MW zinc peaks (Fig. 3B), without a change in the overall distribution of these zinc peaks (Fig. S3, ESI†). Conversely, the abundance of zinc in the low MW pool was increased in the extracts from copper-treated cells, potentially indicating a re-distribution of zinc in copper-treated cells (Fig. 3B).

We also detected a single abundant high MW manganese species in the control lysate (Fig. 3A and Fig. S4A, ESI†). Manganese generally associates only weakly with metalloproteins, and thus is anticipated to dissociate during chromatography, except in proteins that kinetically trap the metal cofactor within a tightly folded protein structure.^26^ We thus reasoned that this manganese pool was likely to be associated with one or both of the Mn/Fe-dependent superoxide dismutases (SODs) produced by *S. aureus*,^36^,^50^ as SODs kinetically trap their cofactor.^51^ Indeed, enzymatic activity was detected from the two *S. aureus* SOD enzymes in the fractions that contained manganese (Fig. S4B, ESI†). SDS-PAGE analysis of eluate fractions from both SEC analysis, using either a single (Fig. S4B, ESI†) or double (Fig. S4C, ESI†) SW3000 column, identified two bands whose intensity was consistent with that of the manganese; one of these was identified by PMF as being the SOD isozyme SodA (9 peptides matched, representing 29% sequence coverage, Mascot expect score: 7.2 × 10^–7^). Comparison of the two-dimensional surface plot of the manganese distribution of a mutant strain (*S. aureus* SH1000 Δ*sodA*Δ*sodM*) unable to produce either of these SODs^50^ confirmed that this high MW manganese peak consisted of the staphylococcal SODs (Fig. S4D, ESI†). No significant change was observed in the manganese distribution in the extracts from cells cultured in high copper (Fig. 3B).

### Identification of a putative copper-associated protein in *S. aureus*

We next sought to determine whether the poorly resolved, high MW copper pool observed in extracts from copper-treated *S. aureus* cells consisted of one or several protein species. We first resolved the AEC fraction previously found to contain the highest levels of copper (Fig. 3B) by a higher resolution HPLC-SEC analysis. This resolved the apparent single copper peak (Fig. 3B) into at least four separate copper-containing species (Fig. S5A, ESI†). Aliquots of the fractions around the most abundant of these copper peaks (Fig. S5B, ESI†) were analysed by SDS-PAGE, and three distinct proteins were found to peak in the same fraction that contained maximal copper (Fig. S5C, ESI†). Each of these three bands were subjected to peptide mass fingerprinting (PMF); two were unable to be identified, but the third was identified as the glyceraldehyde-3-phosphate dehydrogenase (GAPDH) enzyme GapA (19 peptides, representing 61% sequence coverage, Mascot expect score: 3.7 × 10^–7^).

**Fig. 4:**
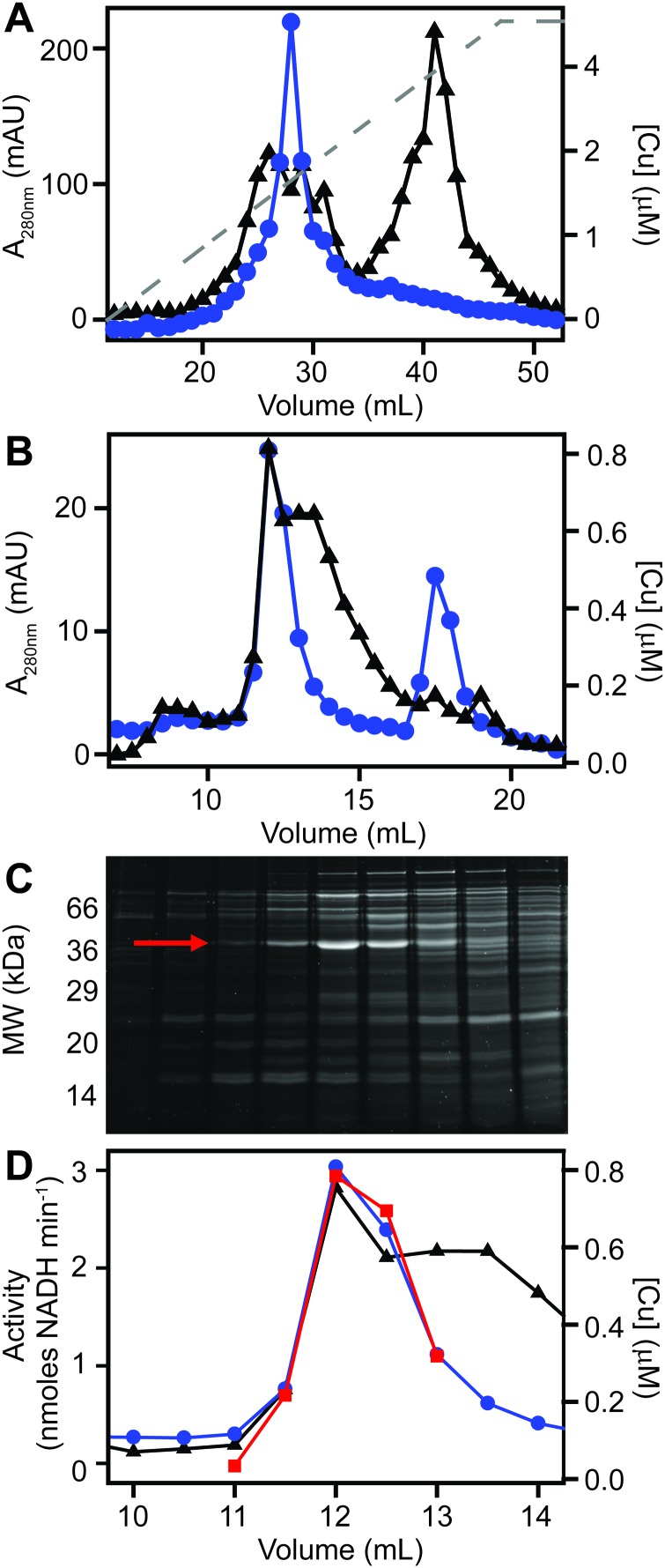
Purification of copper-associated GapA from extracts of copper-exposed *S. aureus* cells. *S. aureus* SH1000 cells (biomass from a total of 2.5 L culture) were cultured in TSB medium containing 50 μM CuSO_4_, harvested and washed as described (see Methods). Cell extract was prepared and resolved by liquid chromatography under anaerobic conditions. The soluble cell fraction was initially passed through a hydroxyapatite column in 5 mM sodium phosphate, pH 6.8 (data not shown), and then the unbound flow-through fraction was buffer exchanged into 10 mM Tris, pH 7.5. (A) This flow-through was subsequently resolved by anion exchange chromatography (AEC) at pH 7.5 on a 1 mL AEC column, eluted with a linear [NaCl] gradient (grey dashed line, 0–1 M NaCl), and fractions analysed for protein content by *A*_280nm_ (black triangles) and for copper (blue circles) by ICP-MS. (B) An aliquot (500 μL) of the peak copper-containing fraction from AEC was subsequently resolved by SEC on a Superdex 200 Increase column in 20 mM Tris, pH 7.5, 150 mM NaCl, analysed for protein by *A*_280nm_ (black) and for copper by ICP-MS (blue). (C) Fractions around the copper peak were analysed by SDS-PAGE, and the protein band identified by peptide mass fingerprinting as GapA is indicated by an arrow. (D) Selected fractions were also assayed for GAPDH activity (red squares), which co-migrated with the copper.

To clarify which proteins were associated with copper in *S. aureus* extracts, we modified the liquid chromatographic separation in an effort to improve the resolution of separation. Using a larger cell pellet from *S. aureus* cells cultured for 16 h in TSB medium to which 50 μM CuSO_4_ was added before inoculation, we prepared anaerobic extract and loaded it on a hydroxyapatite column under anaerobic conditions for initial purification. None of the copper-containing species appeared to bind to the column, with the eluate containing the same copper concentration as the sample loaded (data not shown), and thus the flow-through fraction from hydroxyapatite was subsequently loaded onto AEC. The AEC column was eluted with a linear [NaCl] gradient, and the resulting fractions were analysed for copper by ICP-MS (Fig. 4A). The peak copper-containing fraction again correlated with ∼400 mM NaCl in the elution buffer. This fraction was subjected to a third dimension of chromatography by SEC, this time using a Superdex 200 matrix, resolving a single high MW copper peak (Fig. 4B). SDS-PAGE analysis demonstrated a single protein whose abundance correlated with copper across these chromatographic fractions (Fig. 4C). This protein was identified by PMF as GapA (5 peptides detected, representing 66% sequence coverage, Mascot expect score: 6 × 10^–10^). To validate the presence of GapA in these chromatographic fractions, aliquots were assayed for GAPDH enzymatic activity using an established assay;^31^ GAPDH activity was found to correlate with the abundance of copper (Fig. 4D).

### GapA binds copper inside *S. aureus* cells cultured in the presence of excess copper

In order to validate the identification of GapA as being copper-associated in *S. aureus* cells cultured in the presence of excess exogenous copper, we first aimed to compare metalloproteomic analyses of wild type *S. aureus* cells with those of a mutant strain (*S. aureus* SH1000 Δ*gapA*) unable to synthesise GapA. We acquired the Δ*gapA* strain of a related *S. aureus* strain,^31^ and transduced^50^ this mutant allele into SH1000 using *S. aureus* phage φ11 (Fig. S6, ESI†). As expected,^31^ the resulting mutant strain was unable to grow in TM medium containing glucose due to catabolite repression, but showed growth equivalent to wild type cells on alternative carbon sources such as pyruvate or amino acids (Fig. S7A, ESI†), and extracts from Δ*gapA* cells were devoid of detectable GAPDH activity (Fig. S7B, ESI†).

**Fig. 5:**
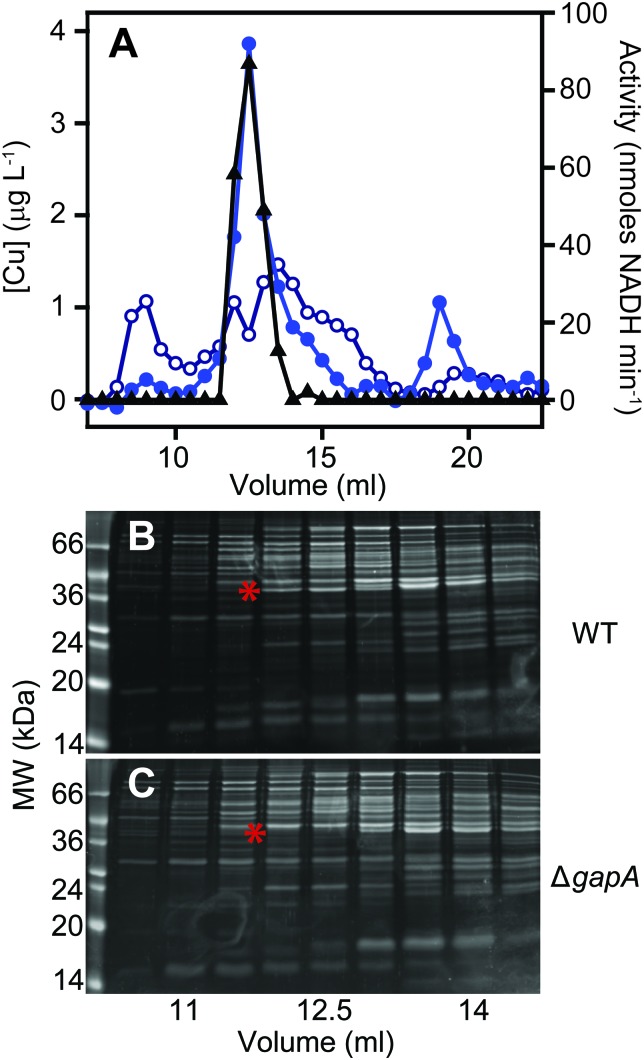
The GapA-associated copper pool is absent in the Δ*gapA* mutant strain. The *S. aureus* SH1000 wild type and the Δ*gapA* mutant strain were cultured in TM medium (0.5 L) with no added glucose but containing 50 μM CuSO_4_. Extracts were prepared and resolved under anaerobic conditions by AEC, and the fraction eluted by 400 mM NaCl from the wild type extract was found to contain most of the GAPDH activity. Aliquots (500 μL) of the 400 mM NaCl AEC fractions from each strain were further resolved by SEC on a Superdex 200 Increase column in 10 mM Tris, 150 mM NaCl, pH 7.5. SEC fractions were analysed for copper (blue circles: closed = WT, open = Δ*gapA* mutant) by ICP-MS and for GAPDH activity using an enzyme assay (black triangles) (A). Note that the SEC fractions from the Δ*gapA* mutant strain were devoid of GAPDH activity (data not shown). SDS-PAGE analysis of selected SEC fractions from both the wild type (B) and the Δ*gapA* mutant (C) confirmed that the protein distribution was essentially unchanged between the two chromatographs, with the exception of the presence of the ∼36 kDa GapA band (highlighted by a red asterisk) in the wild type fractions that was absent in the Δ*gapA* mutant samples (also indicated with a red asterisk).

We prepared extracts from paired cultures of *S. aureus* SH1000 wild type and Δ*gapA* cells cultured in TM medium to which 50 μM CuSO_4_ was added prior to inoculation. It should be noted that in this experiment, the TM minimal medium lacking glucose was used to ensure robust growth of the Δ*gapA* strain (Fig. S7A, ESI†). This copper concentration did not inhibit the growth of the Δ*gapA* strain (data not shown), consistent with the observation that low copper doses had no effect on the growth of either strain (Fig. S7C, ESI†). Cells were harvested after 6 h of growth, and lysates prepared anaerobically. Each extract was resolved identically by AEC under anaerobic conditions, eluted with stepwise increases in NaCl concentration, and the eluate analysed for GAPDH activity using the *in vitro* assay (data not shown). The fraction eluted from AEC by 400 mM NaCl of wild type extract was found to contain maximal GAPDH activity, and therefore each AEC 400 mM NaCl fraction, from WT and Δ*gapA* extracts, was resolved by SEC on Superdex 200. ICP-MS analysis of the SEC eluate demonstrated an abundant copper peak in WT extracts that was absent in the eluate in the Δ*gapA* extract SEC, which co-migrated with detectable GAPDH activity in the WT extract (Fig. 5A). SDS-PAGE analysis of the SEC fractions from the WT extract (Fig. 5B) demonstrated the presence of the GapA protein in the fractions that contained both copper and detectable GAPDH activity, but which was absent in the same fractions, which also lacked copper, in the Δ*gapA* extract (Fig. 5C). No other major changes in protein distribution in these fractions between WT and Δ*gapA* extracts were observed (Fig. 5). From these metalloproteomic data we conclude that the cytosolic enzyme GapA associates with copper in *S. aureus* cells during culture in the presence of elevated concentrations of exogenous copper.

### Binding of copper to *S. aureus* GapA inhibits GAPDH activity

The family of GAPDH enzymes are amongst the most extensively studied enzymes in history, but no member of this family has been reported to require a metal ion for function. Indeed, GAPDH enzymes can be inhibited by divalent metal ions *in vitro*, including Cu(ii).^52^,^53^ To determine whether the binding of copper to GapA detected in metalloproteomic analysis of *S. aureus* extracts had affected its enzymatic activity, we first tested the GAPDH activity in the fractions from SEC after incubation with the copper chelator bathocuproine disulfonate (BCS) and found that this resulted in an increase in detectable GAPDH activity (Fig. S8A, ESI†). BCS specifically chelates the reduced, cuprous ion with high affinity.^54^ We thus tested whether anaerobic incubation of the peak GAPDH-containing fraction with Cu(i) inhibited activity, and found that activity was completely inhibited by addition of 10 μM Cu(i) (Fig. S8B, ESI†).

**Fig. 6:**
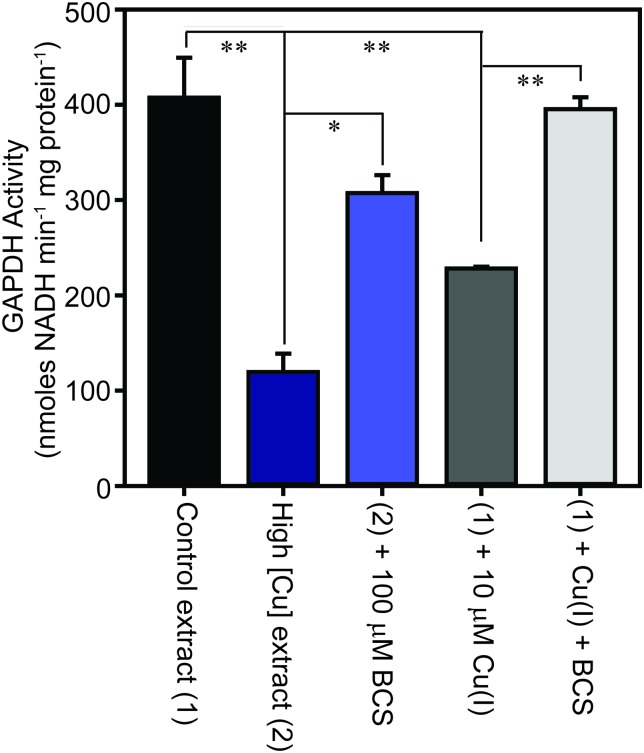
*S. aureus* cells cultured in the presence of copper exhibit reduced GAPDH activity. *S. aureus* SH1000 cells were cultured in TSB medium in the absence (control sample – 1, black) or presence (high [Cu] sample – 2, dark blue) of 0.5 mM CuSO_4_. The cells were washed extensively in EDTA, and then lysed in the absence of EDTA, and the extracts assayed for GAPDH activity, demonstrating a reduction in enzymatic activity in cells cultured in the presence of elevated copper. The lysate that was prepared from cells cultured in the presence of copper was subsequently treated *in vitro* with a copper chelator, through anaerobic incubation of the extract for 10 min in the presence of 100 μM BCS (light blue), which resulted in increased GAPDH activity, implying Cu(i) chelation had alleviated GAPDH inhibition. Likewise, anaerobic incubation of the control lysate with 10 μM Cu(i) (dark grey) reduced the activity, which was reversed on subsequent addition of BCS (light grey).

To verify whether binding of Cu(i) inhibits catalysis by *S. aureus* GapA *in vivo*, we prepared extracts from *S. aureus* SH1000 cells cultured in the absence or presence of copper in TSB medium and assayed them for GAPDH activity (Fig. 6). We found that cells cultured in high copper exhibited reduced GAPDH activity, and that this latter sample's activity was increased to close to control levels by anaerobic incubation of the protein extract with BCS prior to the enzyme assay. Conversely, addition of Cu(i) anaerobically to the control lysate, prepared from cells cultured in the absence of copper, resulted in diminished GAPDH activity, an effect that was reversed by addition of BCS (Fig. 6). In an analogous experiment, we found that GAPDH activity detectable in *S. aureus* cell lysates was also strongly reduced after the addition of monovalent silver ions, which commonly bind to biological Cu(i) binding sites, but was unaffected after addition of divalent cobalt to the lysates, whereas addition of zinc ions (or to a lesser extent nickel ions, which did not reach statistical significance), resulted in intermediate levels of inhibition of enzyme activity (Fig. S9, ESI†).

The observed reduction in detectable GAPDH activity in *S. aureus* cells cultured in the presence of copper was not due to reduced GapA expression under these conditions. Our proteomics data demonstrated that the abundance of GapA is increased, not decreased, in cells cultured in the presence of excess copper. Reverse transcription quantitative PCR (RT-qPCR) analysis also demonstrated no statistically significant change in abundance of the *gapA* transcript in cells cultured in high copper, relative to control cells cultured in low copper (copper-treated cells contained 1.23 ± 0.39 times that detected in control cells), whereas the known copper-regulated gene, *copA*, showed substantial induction under these same conditions (data not shown). We also observed that constitutive over-expression of the *gapA* gene from a plasmid in wild type cells did not increase copper tolerance in TM medium containing glucose (data not shown).

Collectively, these data suggest that in *S. aureus* cells cultured in the presence of high copper, the GapA enzyme becomes partially associated with Cu(i), the form of copper that is anticipated to predominate in the reducing conditions of the staphylococcal cytosol. This binding of Cu(i) inhibits the catalytic activity of GapA. Given that GAPDH is the rate-limiting catalytic step in glycolysis, our conclusion is that excess copper likely inhibits flux through this metabolic pathway. The metabolic versatility of *S. aureus* apparently enables it to continue to utilise glucose (which it must, due to carbon catabolite repression) under conditions of relatively high copper, as growth continues even in the presence of high copper concentrations (Fig. 1). This may be due to the inherent high level of expression of GapA (which was the most abundant protein detected in *S. aureus* extracts), or through the induction by copper of GapA (Tables 1 and 2) or the induction of other steps in glycolysis (Tables 1–4) to optimise glycolytic flux under copper stress conditions.

## Discussion

Copper toxicity is a continuous selection pressure on bacteria due to its ubiquitous presence in the environment, and also due to its use as a weapon in the antimicrobial arsenal of grazing protozoa and of phagocytic cells of the immune system.^13^,^14^,^17^,^55^ The inherent antimicrobial properties of copper are also exploited by mankind in diverse medical and agricultural products. Conversely, copper is also an essential micronutrient for most organisms, therefore bacteria have evolved to balance their need for copper acquisition to supply important copper-dependent enzyme against copper detoxification to prevent toxicity.

The Gram positive staphylococci possess a conserved copper detoxification system that protects against copper intoxication.^32^,^34^,^35^ It is unclear what concentrations of exogenous copper are experienced by bacteria, either during a commensal lifestyle or during infection. Recent data, however, have suggested that the acquisition of additional copper detoxification system by strains of *S. aureus* may have been crucial in the recent evolution and spread of new, highly virulent strains of MRSA that are responsible for current epidemics in North and South America.^9^–^11^,^56^ These newly acquired copper hyper-resistance genes have been integrated into the core copper regulon, as they are regulated by the native *S. aureus* copper-sensing transcriptional regulator CsoR.^10^,^11^ For this reason, it's important to determine how excess copper impacts on *S. aureus* physiology, and how this bacterium adapts to growth in the presence of excess copper in order to understand how these strains have evolved, how they may evolve and spread in the future, and to develop new therapeutic strategies with which to treat them clinically.

Our proteomic analysis has elucidated the adaptive changes that enable *S. aureus* cells to grow in the presence of excess copper concentrations. Crucially, the precise nature of the adaptive changes observed differed between cells exposed to copper excess in a rich *versus* a minimal growth medium (Tables 1–4), demonstrating that the nature of bacterial adaptation to copper stress is dependent on other aspects of their metabolism. This might reflect differential vulnerability to copper toxicity of the specific enzymes involved in different metabolic pathways, or alternatively may be a reflection of the distinct chemical composition of the cytosol under different growth regimes. This latter possibility can be considered analogous to the observation that the extent of copper toxicity experienced by bacteria is conditional on the composition of the growth medium (Fig. 1), mediated by more extensive complexation of the copper in rich media, thereby reducing the effective metal bioavailability.

As has been noted in previous expression analyses,^5^,^57^ a relatively small number of significant changes in expression are necessary and sufficient to enable the bacteria to endure the presence of excess copper. The predominant response to copper stress involved the high-level induction of the conserved copper detoxification system, CopAZ (Tables 1–4), which together act to efflux copper from the cytosol.^35^ This copper-specific cellular response can be considered the ‘core’ copper regulon of *S. aureus*, which is regulated by CsoR.^32^,^34^ These changes were, however, accompanied by a number of more general stress responses. It is currently unclear whether these apparently non-specific responses are mediated directly by the action of cytosolic copper ions on existing regulatory networks, or whether these regulons were induced by transcription factors that are insensitive to copper but are responsive to stress signals experienced by *S. aureus* during exposure to copper. Future studies should aim to combine quantitative assessment of transcript and protein abundance at multiple copper doses to elucidate the hierarchy of regulatory networks, both transcriptional and post-transcriptional, that are critical to *S. aureus* resistance to copper stress.^58^ We observed expression changes in enzymes involved in the oxidative stress response, suggesting cells exposed to high concentrations of copper experience elevated ROS formation, and changes in abundance of proteins involved in transcription and translation, suggesting these fundamental process are affected by cellular copper stress. We also observed indicators of envelope stress, with adaptive changes in expression of enzymes involved in fatty acid biosynthesis implying deficient lipid biosynthesis or adaptive changes in lipid composition, as well as induction of an enzyme of the Dlt system, which is known to modify the cell wall structure to reduce its overall negative charge.^43^ We propose that the latter adaptation functions to reduce the association of copper ions with the cell wall to limit copper exposure and uptake. Future experiments will aim to elucidate the function of this cell wall modification in adaptation to copper stress and to determine whether these changes to wall structure make *S. aureus* cells more vulnerable to killing by CAMPs.

A clear observation from our proteomics analysis was that *S. aureus* adapts to copper stress by modifying the expression of a number of enzymes involved in central carbon metabolism. Specifically, cells cultured under all four tested high-copper growth conditions exhibited differential expression of enzymes involved in glycolysis (Fda, GapA, GpmA – Tables 1–4), consistent with adaptation to copper stress requiring *S. aureus* to optimise its abundance of glycolytic enzymes to maintain flux through this core glucose-metabolising pathway. Other adaptive changes observed involved down-regulation of enzymes that metabolise pyruvate (Tables 1–4), further demonstrating how carbon utilisation is modified under copper stress. The *gapA* gene is known to be regulated by GapR,^31^ which is hypothesised to sense the abundance of fructose-1,6-bisphosphate,^59^ as well as the carbon catabolite regulator CcpA,^60^ which mediates overall control over carbon utilization. Conversely, no putative binding site for the copper sensor CsoR could be detected upstream of the promoter from which *gapA* is expressed. Thus any copper-mediated transcriptional regulation of *gapA* expression is likely to be indirect. A future study should aim to elucidate how these adaptations of *S. aureus* carbon metabolism enable the bacterium to grow in the presence of high exogenous copper concentrations, and the regulatory mechanisms that determine how these adaptive changes are mediated by copper within *S. aureus* cells.

We subsequently studied how the increased cellular copper that is accumulated by *S. aureus* cells cultured in the presence of high concentrations of copper (Fig. 2) is distributed within the cells. We observed that cytosolic extracts from those cells also exhibited increased copper accumulation, which after fractionation was clearly associated with proteinaceous species (Fig. 3). Using established approaches, we identified one of these protein species with which copper associates in cells cultured in the presence of excess copper as the glycolytic glyceraldehyde-3-phosphate dehydrogenase enzyme, GapA (Fig. 4 and 5). This association of GapA with copper leads to inhibition of its enzyme activity, suggesting a potential mechanism by which the adaptive changes observed in the abundance of glycolytic enzymes (Tables 1–4) is mediated. We propose that decreased flux through glycolysis, caused by copper-dependent inhibition of its rate-limiting step, GAPDH, is sensed by the cell and then fed back to increase expression of potentially blocked steps, including of GapA itself. Future studies will aim to elucidate (i) the mechanism by which binding of copper to GapA inhibits activity, (ii) how this inhibition is sensed by *S. aureus*, and how its induction of glycolytic enzymes is mediated, and (iii) how the observed adaptive changes in enzyme expression are sufficient to enable *S. aureus* to grow robustly in the presence of high concentrations of copper.

## Conflicts of interest

There are no conflicts to declare.

## Supplementary Material

Supplementary information

Supplementary information

## References

[cit1] Djoko K. Y., McEwan A. G. (2013). ACS Chem. Biol..

[cit2] Macomber L., Imlay J. A. (2009). Proc. Natl. Acad. Sci. U. S. A..

[cit3] Barwinska-Sendra A., Waldron K. J. (2017). Adv. Microb. Physiol..

[cit4] Macomber L., Rensing C., Imlay J. A. (2007). J. Bacteriol..

[cit5] Baker J., Sitthisak S., Sengupta M., Johnson M., Jayaswal R. K., Morrissey J. A. (2010). Appl. Environ. Microbiol..

[cit6] da Silva J. J. R. F. and Williams R. J. P., The Biological Chemistry of the Elements: The Inorganic Chemistry of Life, OUP, Oxford, 2001.

[cit7] Bull P. C., Thomas G. R., Rommens J. M., Forbes J. R., Cox D. W. (1993). Nat. Genet..

[cit8] Chelly J., Tumer Z., Tonnesen T., Petterson A., Ishikawa-Brush Y., Tommerup N., Horn N., Monaco A. P. (1993). Nat. Genet..

[cit9] Planet P. J., Diaz L., Kolokotronis S. O., Narechania A., Reyes J., Xing G., Rincon S., Smith H., Panesso D., Ryan C., Smith D. P., Guzman M., Zurita J., Sebra R., Deikus G., Nolan R. L., Tenover F. C., Weinstock G. M., Robinson D. A., Arias C. A. (2015). J. Infect. Dis..

[cit10] Purves J., Thomas J., Riboldi G. P., Zapotoczna M., Tarrant E., Andrew P. W., Londono A., Planet P. J., Geoghegan J. A., Waldron K. J., Morrissey J. A. (2018). Environ. Microbiol..

[cit11] Zapotoczna M., Riboldi G. P., Moustafa A. M., Dickson E., Narechania A., Morrissey J. A., Planet P. J., Holden M. T. G., Waldron K. J., Geoghegan J. A. (2018). mBio.

[cit12] Harkins C. P., Pichon B., Doumith M., Parkhill J., Westh H., Tomasz A., de Lencastre H., Bentley S. D., Kearns A. M., Holden M. T. G. (2017). Genome Biol..

[cit13] Hyre A. N., Kavanagh K., Kock N. D., Donati G. L., Subashchandrabose S. (2017). Infect. Immun..

[cit14] White C., Lee J., Kambe T., Fritsche K., Petris M. J. (2009). J. Biol. Chem..

[cit15] Francis M. S., Thomas C. J. (1997). Microb. Pathog..

[cit16] Johnson M. D., Kehl-Fie T. E., Klein R., Kelly J., Burnham C., Mann B., Rosch J. W. (2015). Infect. Immun..

[cit17] Ladomersky E., Khan A., Shanbhag V., Cavet J. S., Chan J., Weisman G. A., Petris M. J. (2017). Infect. Immun..

[cit18] Shafeeq S., Yesilkaya H., Kloosterman T. G., Narayanan G., Wandel M., Andrew P. W., Kuipers O. P., Morrissey J. A. (2011). Mol. Microbiol..

[cit19] Malachowa N., Whitney A. R., Kobayashi S. D., Sturdevant D. E., Kennedy A. D., Braughton K. R., Shabb D. W., Diep B. A., Chambers H. F., Otto M., DeLeo F. R. (2011). PLoS One.

[cit20] Noyce J. O., Michels H., Keevil C. W. (2006). J. Hosp. Infect..

[cit21] Casey A. L., Adams D., Karpanen T. J., Lambert P. A., Cookson B. D., Nightingale P., Miruszenko L., Shillam R., Christian P., Elliott T. S. J. (2010). J. Hosp. Infect..

[cit22] Palza H., Nuñez M., Bastías R., Delgado K. (2018). Int. J. Antimicrob. Agents.

[cit23] Horsburgh M. J., Aish J. L., White I. J., Shaw L., Lithgow J. K., Foster S. J. (2002). J. Bacteriol..

[cit24] Sebulsky M. T., Hohnstein D., Hunter M. D., Heinrichs D. E. (2000). J. Bacteriol..

[cit25] Townsend D. E., Wilkinson B. J. (1992). J. Bacteriol..

[cit26] Tottey S., Waldron K. J., Firbank S. J., Reale B., Bessant C., Sato K., Cheek T. R., Gray J., Banfield M. J., Dennison C., Robinson N. J. (2008). Nature.

[cit27] Tu W. Y., Pohl S., Gray J., Robinson N. J., Harwood C. R., Waldron K. J. (2012). J. Bacteriol..

[cit28] Beauchamp C., Fridovich I. (1971). Anal. Biochem..

[cit29] Vita N., Platsaki S., Basle A., Allen S. J., Paterson N. G., Crombie A. T., Murrell J. C., Waldron K. J., Dennison C. (2015). Nature.

[cit30] Morrissey J. A., Cockayne A., Hill P. J., Williams P. (2000). Infect. Immun..

[cit31] Purves J., Cockayne A., Moody P. C., Morrissey J. A. (2010). Infect. Immun..

[cit32] Baker J., Sengupta M., Jayaswal R. K., Morrissey J. A. (2011). Environ. Microbiol..

[cit33] Rathnayake I. V. N., Megharaj M., Krishnamurti G. S. R., Bolan N. S., Naidu R. (2013). Chemosphere.

[cit34] Grossoehme N., Kehl-Fie T. E., Ma Z., Adams K. W., Cowart D. M., Scott R. A., Skaar E. P., Giedroc D. P. (2011). J. Biol. Chem..

[cit35] Sitthisak S., Knutsson L., Webb J. W., Jayaswal R. K. (2007). Microbiology.

[cit36] Garcia Y. M., Barwinska-Sendra A., Tarrant E., Skaar E. P., Waldron K. J., Kehl-Fie T. E. (2017). PLoS Pathog..

[cit37] Horsburgh M. J., Wharton S. J., Cox A. G., Ingham E., Peacock S., Foster S. J. (2002). Mol. Microbiol..

[cit38] Pother D. C., Gierok P., Harms M., Mostertz J., Hochgrafe F., Antelmann H., Hamilton C. J., Borovok I., Lalk M., Aharonowitz Y., Hecker M. (2013). Int. J. Med. Microbiol..

[cit39] Peng H., Zhang Y., Palmer L. D., Kehl-Fie T. E., Skaar E. P., Trinidad J. C., Giedroc D. P. (2017). ACS Infect. Dis..

[cit40] Bischoff M., Dunman P., Kormanec J., Macapagal D., Murphy E., Mounts W., Berger-Bachi B., Projan S. (2004). J. Bacteriol..

[cit41] Pförtner H., Burian M. S., Michalik S., Depke M., Hildebrandt P., Dhople V. M., Pané-Farré J., Hecker M., Schmidt F., Völker U. (2014). Int. J. Med. Microbiol..

[cit42] Tuchscherr L., Bischoff M., Lattar S. M., Noto Llana M., Pfortner H., Niemann S., Geraci J., Van de Vyver H., Fraunholz M. J., Cheung A. L., Herrmann M., Volker U., Sordelli D. O., Peters G., Loffler B. (2015). PLoS Pathog..

[cit43] Peschel A., Otto M., Jack R. W., Kalbacher H., Jung G., Gotz F. (1999). J. Biol. Chem..

[cit44] Muller M., Reiss S., Schluter R., Mader U., Beyer A., Reiss W., Marles-Wright J., Lewis R. J., Pfortner H., Volker U., Riedel K., Hecker M., Engelmann S., Pane-Farre J. (2014). Mol. Microbiol..

[cit45] Monteiro J. M., Fernandes P. B., Vaz F., Pereira A. R., Tavares A. C., Ferreira M. T., Pereira P. M., Veiga H., Kuru E., VanNieuwenhze M. S., Brun Y. V., Filipe S. R., Pinho M. G. (2015). Nat. Commun..

[cit46] Tottey S., Patterson C. J., Banci L., Bertini I., Felli I. C., Pavelkova A., Dainty S. J., Pernil R., Waldron K. J., Foster A. W., Robinson N. J. (2012). Proc. Natl. Acad. Sci. U. S. A..

[cit47] Osman D., Waldron K. J., Denton H., Taylor C. M., Grant A. J., Mastroeni P., Robinson N. J., Cavet J. S. (2010). J. Biol. Chem..

[cit48] Waldron K. J., Tottey S., Yanagisawa S., Dennison C., Robinson N. J. (2007). J. Biol. Chem..

[cit49] Changela A., Chen K., Xue Y., Holschen J., Outten C. E., O'Halloran T. V., Mondragon A. (2003). Science.

[cit50] Karavolos M. H., Horsburgh M. J., Ingham E., Foster S. J. (2003). Microbiology.

[cit51] Whittaker M. M., Mizuno K., Bachinger H. P., Whittaker J. W. (2006). Biophys. J..

[cit52] Krotkiewska B., Banas T. (1992). Int. J. Biochem..

[cit53] Mounaji K., Vlassi M., Erraiss N. E., Wegnez M., Serrano A., Soukri A. (2003). Comp. Biochem. Physiol., Part B: Biochem. Mol. Biol..

[cit54] Xiao Z., Wedd A. G. (2010). Nat. Prod. Rep..

[cit55] Hao X., Luthje F., Ronn R., German N. A., Li X., Huang F., Kisaka J., Huffman D., Alwathnani H. A., Zhu Y. G., Rensing C. (2016). Mol. Microbiol..

[cit56] Gomez-Sanz E., Kadlec K., Fessler A. T., Zarazaga M., Torres C., Schwarz S. (2013). Antimicrob. Agents Chemother..

[cit57] Kershaw C. J., Brown N. L., Constantinidou C., Patel M. D., Hobman J. L. (2005). Microbiology.

[cit58] Latorre M., Galloway-Pena J., Roh J. H., Budinich M., Reyes-Jara A., Murray B. E., Maass A., Gonzalez M. (2014). Metallomics.

[cit59] Doan T., Aymerich S. (2003). Mol. Microbiol..

[cit60] Ludwig H., Rebhan N., Blencke H. M., Merzbacher M., Stulke J. (2002). Mol. Microbiol..

