# Supplementary material for: Copper stress in Staphylococcus aureus leads to adaptive changes in central carbon metabolism
Source: Metallomics. 2018 Nov 16;11(1):183–200. doi: 10.1039/c8mt00239h (PMC6350627; doi:10.1039/c8mt00239h)
Supplement: Supplementary file 1 [file MT-011-C8MT00239H-s001.pdf]

Electronic Supplementary Material (ESI) for Metallomics.
This journal is © The Royal Society of Chemistry 2018

SUPPLEMENTARY INFORMATION

Copper stress in *Staphylococcus aureus* leads to adaptive changes in central carbon metabolism

Emma Tarrant[1], Gustavo P. Riboldi[1], Matthew R. McIlvin[2], Jack Stevenson[1], Anna Barwinska-Sendra[1], Louisa J. Stewart[1], Mak A. Saito[2], Kevin J. Waldron[1*]

Affiliations:
[1]Institute for Cell & Molecular Biosciences, Faculty of Medical Sciences, Newcastle University, Framlington Place, Newcastle upon Tyne, NE2 4HH, United Kingdom.
[2]Marine Chemistry and Geochemistry Department, Woods Hole Oceanographic Institution, Woods Hole, MA 02543, USA.

*Corresponding Author:
Dr. Kevin J. Waldron. email: kevin.waldron@ncl.ac.uk. Tel: 0191 208 7369. Fax: 0191 208 7424. Postal Address: Dr. Kevin Waldron, Institute for Cell & Molecular Biosciences, Faculty of Medical Sciences, Newcastle University, Framlington Place, Newcastle upon Tyne, NE2 4HH, United Kingdom.

Supplementary Information contents:

Supplementary Figures 1-9: pages 2-12.

References

1. M. H. Karavolos, M. J. Horsburgh, E. Ingham and S. J. Foster, Role and regulation of the superoxide dismutases of *Staphylococcus aureus*, *Microbiology (Reading, England)*, 2003, **149**, 2749-2758.
2. J. Purves, A. Cockayne, P. C. Moody and J. A. Morrissey, Comparison of the regulation, metabolic functions, and roles in virulence of the glyceraldehyde-3-phosphate dehydrogenase homologues *gapA* and *gapB* in *Staphylococcus aureus*, *Infection and immunity*, 2010, **78**, 5223-5232.
3. M. J. Horsburgh, J. L. Aish, I. J. White, L. Shaw, J. K. Lithgow and S. J. Foster, sigmaB modulates virulence determinant expression and stress resistance: characterization of a functional *rsbU* strain derived from *Staphylococcus aureus* 8325-4, *Journal of bacteriology*, 2002, **184**, 5457-5467.

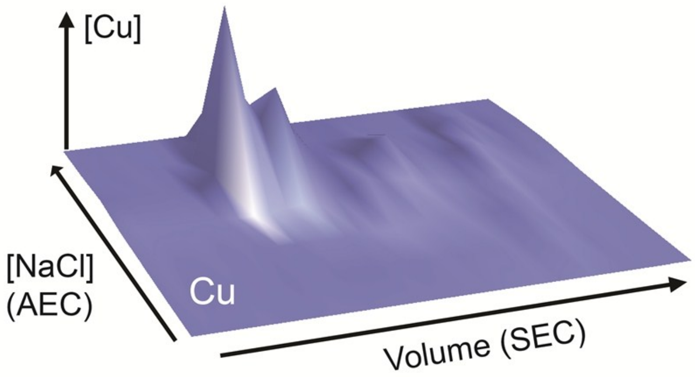

Supplementary Figure 1: Soluble copper pools are present in *S. aureus* cytosolic extracts. An expanded view of the data shown in Fig. 3A on an expanded z-axis (shown between 0 - 0.2 μM Cu here, compared to 0 - 11 μM Cu in Fig. 3A to allow direct comparison to the data from copper-treated cell lysates), demonstrating the presence of high molecular weight copper pools in cytosolic extracts prepared from *S. aureus* cells cultured in standard TSB medium without copper supplementation. The protein species associated with these copper pools are as yet unidentified.

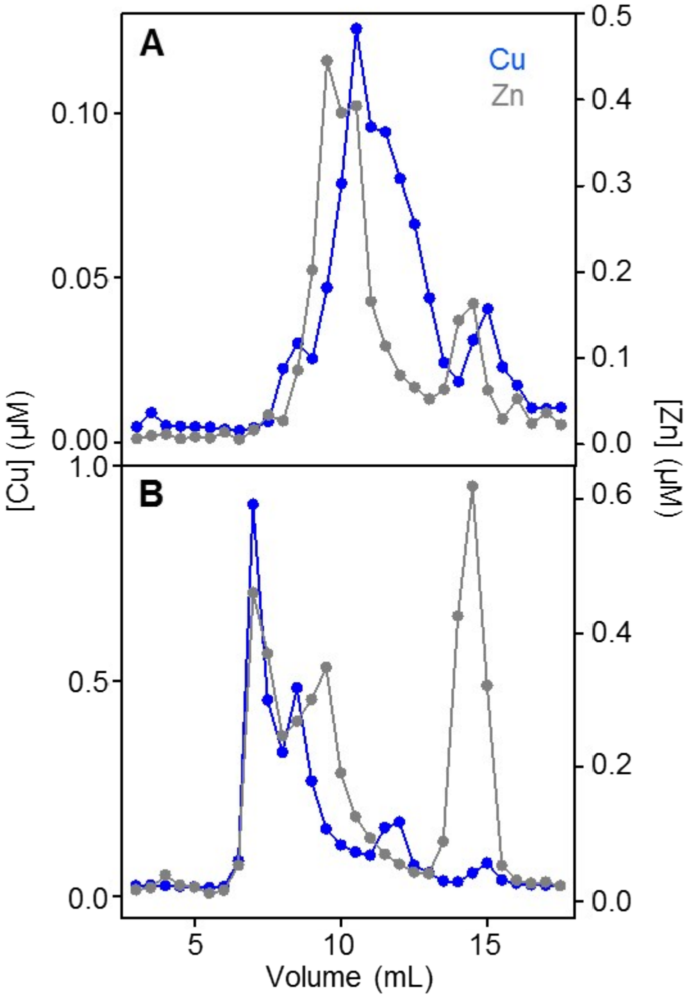

Supplementary Figure 2: Soluble copper and zinc pools present in *S. aureus* extracts are discrete.

An expanded view of a subset of the data shown in Fig. 3A, showing the copper (blue) and zinc (grey) detected in fractions of eluant from the full SEC chromatogram, after resolution of the fraction of eluant eluted from AEC by (A) 300 mM, and (B) 400 mM NaCl, of the control lysate prepared from cells cultured in TSB without copper supplementation. These data demonstrate that most of the zinc peaks do not co-migrate with the copper peaks, implying these two metals are associated with different proteins in the *S. aureus* extracts.

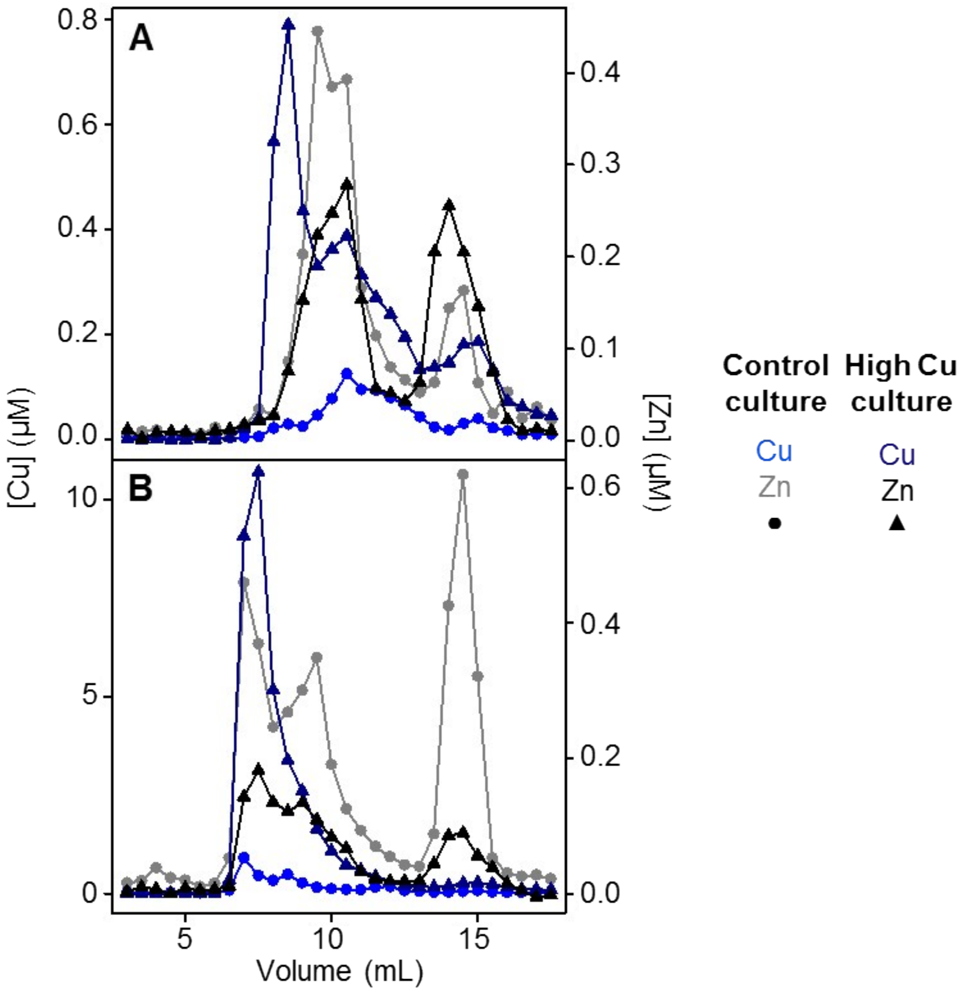

Supplementary Figure 3: Soluble copper and zinc pools present in *S. aureus* extracts are discrete.

A comparison of the distribution of copper (light/dark blue) and zinc (grey/black) detected by ICP-MS in SEC fractions after resolution of the fractions eluted from AEC by (A) 300 mM, and (B) 400 mM of the *S. aureus* extracts, as presented in Fig. 3. The copper (light blue circles) and zinc (grey circles) detected in the fractions derived from the control lysate, prepared from *S. aureus* cells cultured in TSB containing no added copper, is reproduced here from Supp. Fig. 2. Overlaid on top of these data are the corresponding data for copper (dark blue triangles) and zinc (black triangles) from the equivalent analyses of the same fractions derived from the copper-treated cell lysates, prepared from *S. aureus* cells cultured in TSB containing 500 μM $CuSO_4$. These data demonstrate that copper supplementation leads to an overall decrease in the quantity of zinc detected in each of the soluble zinc pools, but does not disturb their overall distribution, implying reduced zinc occupancy of the same protein species under elevated copper growth conditions.

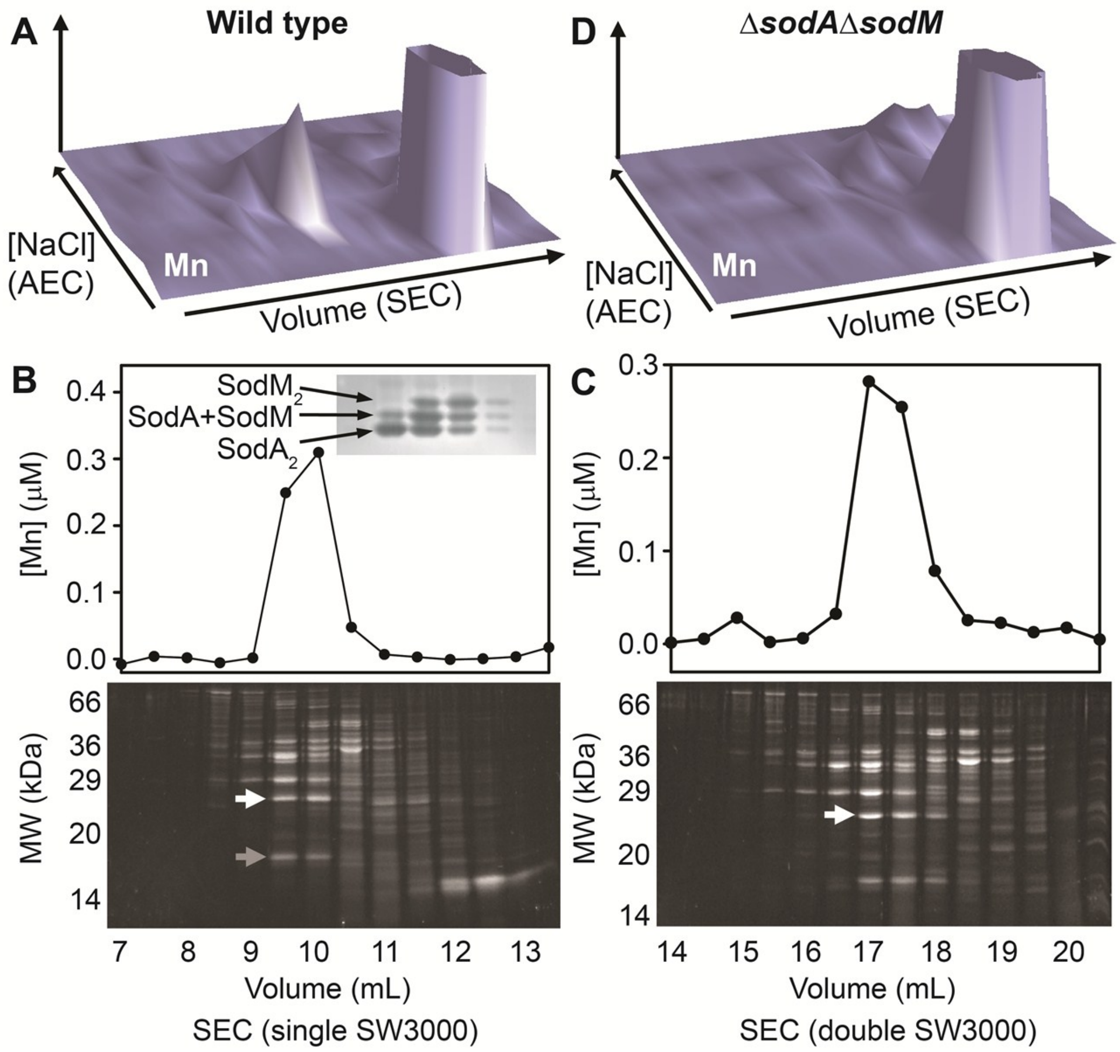

Supplementary Figure 4: The high MW manganese peak detected in *S. aureus* cytosolic extracts are associated with the staphylococcal SOD enzymes.

(A) Extracts of wild type *S. aureus* SH1000 (100 mL cultures in TSB) were anaerobically prepared and resolved by two dimensional liquid chromatography by AEX (1 mL Q HP column, pH 8.5) followed by SEC (SW3000 column, pH 7.5), and the resulting fractions analysed by ICP-MS as described in Methods. A single high MW Mn pool was detected (Mn^{HMW}), as was a single low MW Mn pool which co-migrated with high concentrations of phosphorus as detected by ICP-MS (data not shown). (B) Fractions from the SEC column across the Mn^{HMW} pool were resolved by SDS-PAGE and stained with Sypro Ruby (lower panel), and densitometry showed that two detected protein bands showed a SEC elution profile that matched the distribution of Mn (labelled with arrows). (C) To improve resolution, the same fraction from AEC was resolved by two SEC (SW3000) columns fitted in series, and the analysis repeated. From this analysis, only a single protein band co-migrated with the Mn (white arrow in B and C). This band was excised from the gel and determined by PMF to contain SodA (see main text). As a result, the fractions from the

previous analysis (panel B) were resolved by native PAGE and stained in-gel for SOD activity assay using an established assay, which demonstrated the presence of both of the *S. aureus* SOD enzyme in the fractions that contained Mn (panel B, *inset*). (D) Extracts of a Δ*sodA*Δ*sodM* strain[1] of *S. aureus* SH1000 was prepared, resolved and analysed identically to that of the WT in panel A, in which the Mn^{HMW} pool was found to be absent.

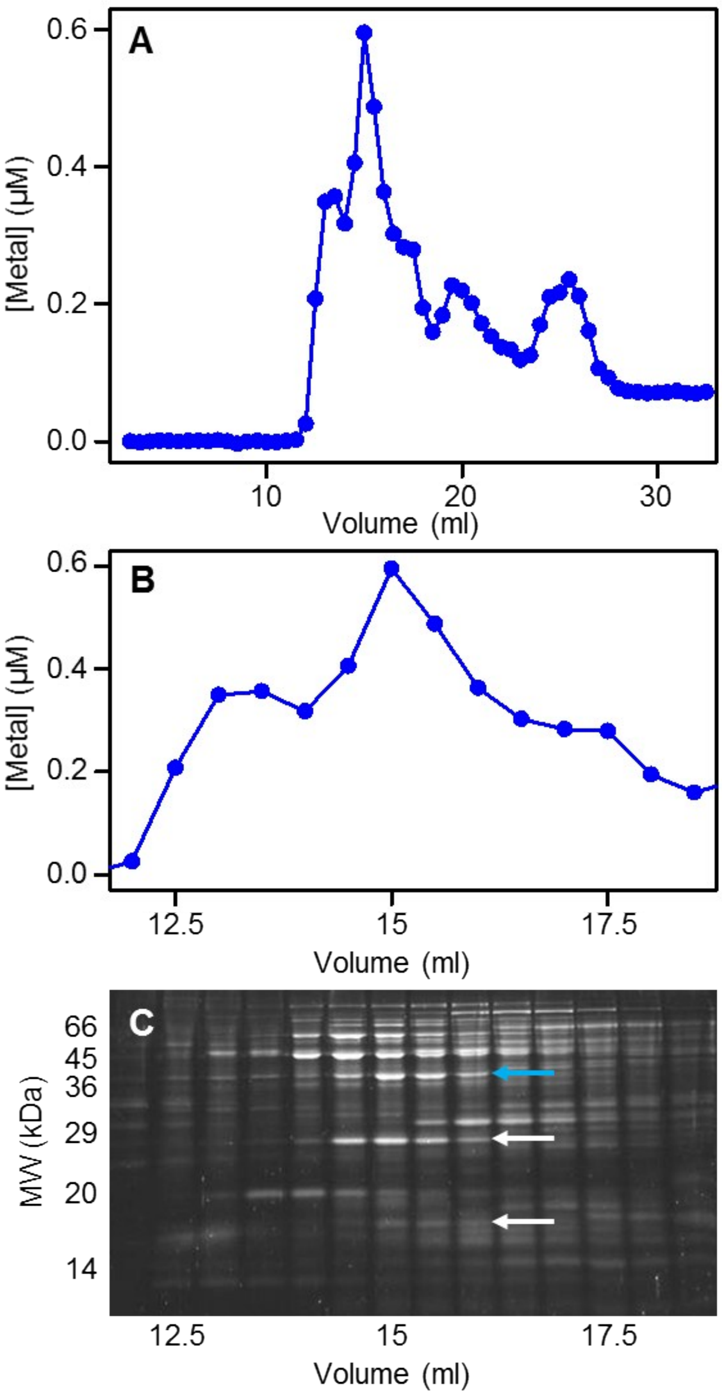

Supplementary Figure 5: Preliminary identification of GapA as a copper-associated protein in extracts from copper-exposed *S. aureus* cells.

S. aureus SH1000 cells were cultured at 37ºC in TSB medium (biomass from a total of total 1 L) containing 0.5 mM $CuSO_4$, harvested and washed (see Methods). Cell extract was prepared and resolved by two dimensional liquid chromatography under anaerobic conditions (Fig. 3B). The soluble cell fraction was purified by anion exchange chromatography (AEX), pH 8.8 (data not shown), and then the fraction eluted with 400 mM NaCl was further resolved by HPLC size exclusion chromatography (SEC) using 2 x SW3000 connected in series, in 5 mM Hepes, pH 7.5, 50 mM NaCl. All fractions were analysed for copper (blue) by ICP-MS (A), and the peak copper-

containing fractions (B) were analysed for protein by SDS-PAGE stained with Sypro Ruby (C). The protein band that was excised from the gel and identified by peptide mass fingerprinting as *S. aureus* GapA is indicated by the blue arrow, and two other potential candidate proteins by white arrows, in panel (C).

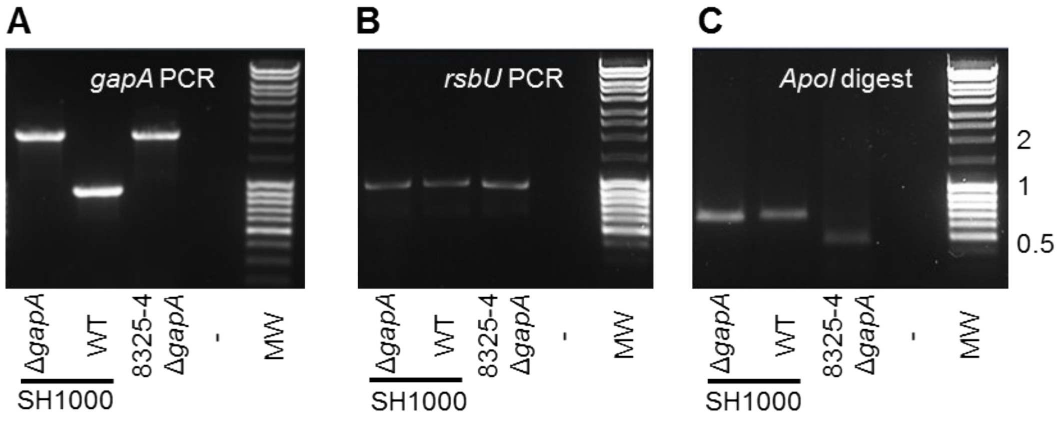

Supplementary Figure 6: Genotype of *S. aureus* SH1000 Δ*gapA* mutant.

The insertionally inactivated *gapA::tet* allele from *S. aureus* 8325-4 Δ*gapA::tet*[2] was transduced using the *S. aureus* φ11 phage into the *S. aureus* SH1000 genetic background[3]. Transductants were selected on tetracycline-containing TM agar plates, genomic DNA prepared and screened by PCR, yielding PCR products of ~1 kb from wild type and ~2 kb from Δ*gapA* on 1% agarose (w/v) gel electrophoresis (A). The recipient SH1000 strain differs from the donor 8325-4 strain from which it was derived by the correction of a mutation that was found in the *rsbU* locus[3]. To verify successful transduction, the *rsbU* gene was amplified by PCR from genomic DNA prepared from all three strains, yielding a ~1 kb PCR product (B), and the wild type *rsbU* status of the SH1000 Δ*gapA* transductant strain confirmed by a diagnostic restriction digest of this product with *Apo*I enzyme, yielding a ~700 bp product from the SH1000 strains and a ~500 bp product from the 8325-4 strain (C). The lane labelled '-' represents a negative control PCR reaction, using water instead of template DNA, and that labelled 'MW' represents molecular weight markers, labelled in kb.

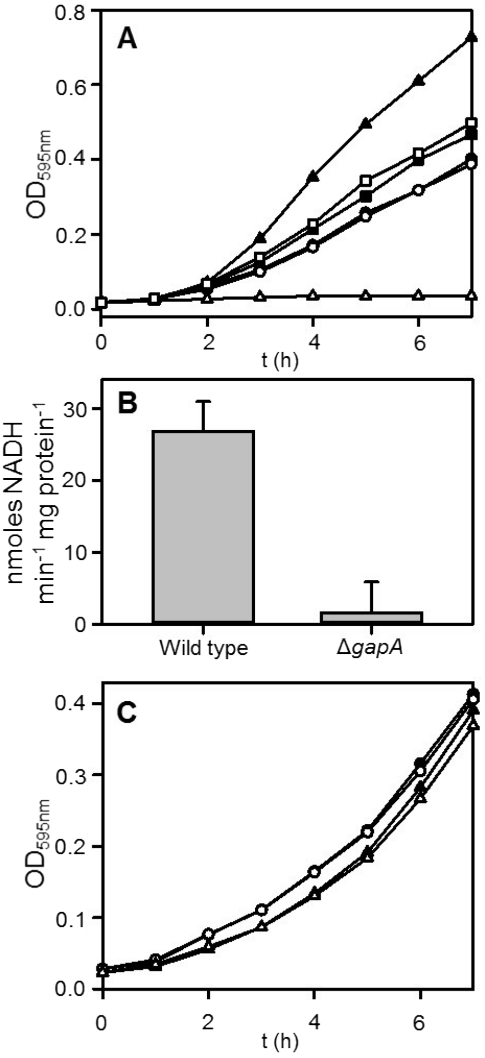

Supplementary Figure 7: The SH1000 Δ*gapA* strain is unable to grow on glucose and exhibits negligible NAD+-dependent GAPDH activity.

(A) Analysis of the growth of wild type *S. aureus* SH1000 (closed symbols) and the SH1000 Δ*gapA* strain (open symbols) in TM medium with no added carbon source (circles) or with 1% glucose (triangles) or 5 mM pyruvate (squares) added. The Δ*gapA* strain failed to grow in the presence of glucose, consistent with a glycolytic defect, but was indistinguishable from wild type when using either pyruvate or amino acids as carbon source, consistent with results from the 8325-4 Δ*gapA* mutant strain[2]. Growth curves were repeated using independent cultures on three separate days, and a representative example is shown. (B) Wild type *S. aureus* SH1000 and the Δ*gapA* mutant strain were cultured in TM medium, cells harvested and washed, lysates prepared and assayed for GAPDH enzyme activity. The Δ*gapA* strain, as expected, contains negligible NAD+-dependent GAPDH activity relative to the wild type, consistent with a previous report using the *S. aureus* 8325-4 strain[2]. (C) Growth of both the wild type (circles) and Δ*gapA* (triangles) *S. aureus* cells in TM lacking any added carbon source is identical in the absence (closed symbols) or presence (open symbols) of 0.25 mM $CuSO_4$.

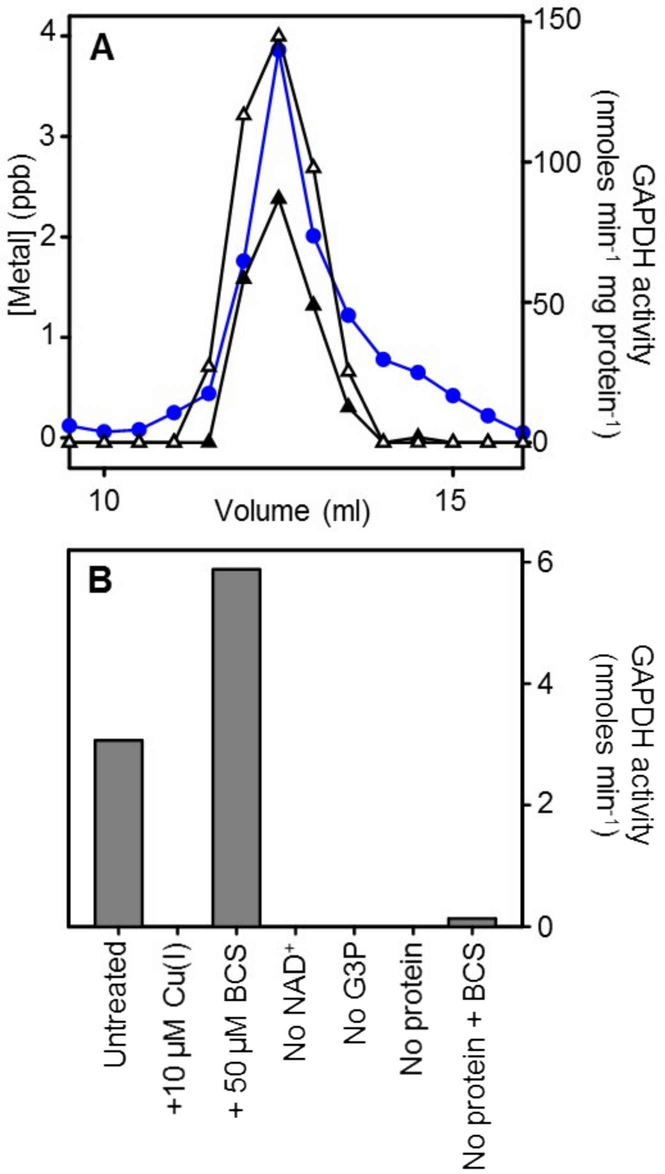

Supplementary Figure 8: Treatment of chromatographic fractions containing *S. aureus* GapA with a copper chelator or with Cu(I) affects enzymatic activity.

(A) The copper- (blue circles) and GapA-containing SEC fractions (Fig. 9) were assayed for GAPDH enzyme activity before (closed triangles) and after (open triangles) 10 min incubation in the presence of 50 µM BCS under anaerobic conditions. Treatment with the copper chelator resulted in an increase in the enzymatic activity detected from the GapA-containing fractions. (B) Enzyme assay of the peak copper- and GapA-containing fraction from SEC (Fig. 5) after 10 min incubation with 10 µM Cu(I) under anaerobic conditions showed elimination of detectable GAPDH activity, whereas incubation with 50 µM BCS led to increased enzyme activity, consistent with panel A. The results from control reactions for the GAPDH enzyme assay are shown here; these controls were performed for all assays in this manuscript (data not shown).

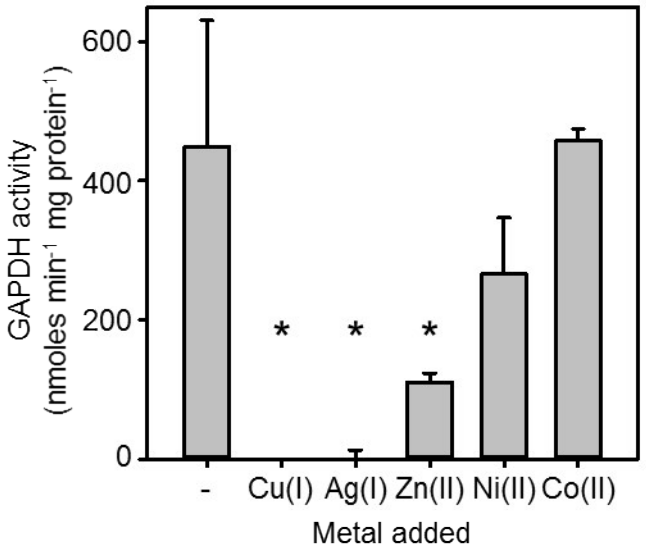

Supplementary Figure 9: Treatment of *S. aureus* lysates with other metal ions has differential effects on GapA enzymatic activity.

(A) Aliquots (15 µg total protein) from a soluble lysate prepared from *S. aureus* SH1000 cells, which had been cultured in TSB medium containing glucose, were assayed for GAPDH enzyme activity after 10 min anaerobic incubation in the presence of 50 µM of each of the metals indicated, or after addition of an equal volume of buffer ('-') as a control. The asterisk represents statistical significance in a student's t-test ($p<0.05$).
